# Single‐Cell Sequencing and Mendelian Randomization Reveal T Cell Nuclear Factor Genes in Hepatocellular Carcinoma Progression

**DOI:** 10.1155/humu/7446280

**Published:** 2026-04-20

**Authors:** Yunfei Chen, Xiting Yang, Lintao Zhong, Kai Chen, Le Luo

**Affiliations:** ^1^ Organ Transplantation Center, Sichuan Provincial People′s Hospital, University of Electronic Science and Technology of China, Chengdu, China, uestc.edu.cn; ^2^ Hepatobiliary and Pancreatic Surgery, Sichuan Provincial People′s Hospital, University of Electronic Science and Technology of China, Chengdu, China, uestc.edu.cn; ^3^ School of Medical and Life Sciences of Chengdu University of Traditional Chinese Medicine, Chengdu, China

**Keywords:** hepatocellular carcinoma, hub gene, Mendelian randomization, nuclear factor of activated T cells, single-cell

## Abstract

This study integrates single‐cell RNA sequencing with Mendelian randomization to elucidate the role of nuclear factor of activated T cells (NFAT)‐related genes in the progression of hepatocellular carcinoma (HCC). The GSE162616 dataset was analyzed to identify differentially expressed cells and NFAT‐related genes through quality control, clustering, and *z*‐score algorithms. Mendelian randomization analysis of expression quantitative trait loci (eQTL) data was performed to identify hub genes causally linked to HCC. Validation in The Cancer Genome Atlas‐Liver Hepatocellular Carcinoma cohort included survival analysis, clinical correlation, and nomogram construction. Sixteen cell clusters were resolved and annotated into five types: natural killer (NK) cells, T cells, B cells, hepatocytes, and monocytes. Differentially expressed NFAT‐related genes were predominantly enriched in immune and cytokine pathways. Three genes—CACYBP, CTLA4, and RGCC—were identified as causally associated with HCC and designated as hub genes. T cells and NK cells emerged as key cellular populations, and pseudotime analysis delineated T cell differentiation trajectories. Cell–cell communication analysis revealed robust interactions between NK and B cells and between NK and T cells, primarily via the MIF–(CD74+CXCR4) axis. All three hub genes were upregulated in HCC tissues. A nomogram integrating these genes exhibited excellent diagnostic performance (AUC = 0.9). These results establish CACYBP and RGCC as risk factors and CTLA4 as a protective factor for HCC. The nomogram offers a potential tool for early diagnosis and immunotherapy guidance. Our findings highlight the value of integrating single‐cell transcriptomics with Mendelian randomization for prioritizing causal genes and provide novel insights into NFAT‐mediated immune regulation in HCC.

## 1. Introduction

Hepatocellular carcinoma (HCC) is the most common form of primary liver cancer and is associated with a high mortality rate. Its etiology is multifactorial, involving viral hepatitis infections (hepatitis B and C viruses), alcoholic and nonalcoholic fatty liver disease (NAFLD), as well as genetic and environmental factors [1–3]. Hepatectomy and liver transplantation remain the mainstay treatments for achieving long‐term survival. In recent years, the combination of targeted therapy and immunotherapy has led to sustained improvements in the objective response rate (ORR) and a significant extension of overall survival (OS) in patients with HCC. Notably, some patients with initially unresectable tumors have become eligible for curative surgery following successful conversion therapy [4]. Despite these advances, the risk of recurrence remains high, with rates reaching up to 70% within 5 years after surgery. Tumor recurrence and metastasis have thus emerged as key determinants of long‐term patient outcomes [5]. Consequently, continued and comprehensive investigation into the molecular mechanisms underlying HCC initiation and progression remains a critical research priority.

The nuclear factor of activated T cells (NFAT) comprises a family of Ca^2+^/calmodulin‐dependent, phosphatase‐responsive transcription factors, classically recognized for their central roles in T lymphocyte activation and cardiac valve development [6]. Accumulating evidence suggests that the NFAT family also contributes to the pathogenesis of various tumor types [7–10]; however, its involvement in liver cancer remains poorly understood. A recent study reported that increased expression of NFATc4 in hepatocytes mediates NAFLD by negatively regulating PPAR*α* and osteopontin, thereby promoting steatosis, hepatic inflammation, and fibrosis [9]. Conversely, Wang et al. [11] demonstrated that the calcineurin/NFATc1 pathway may drive HCC cell proliferation through transcriptional activation of oncogenes such as c‐myc and COX‐2. In 2019, Iyer et al. [12] found that high‐dose sorafenib activates NFAT1, which in turn suppresses T cell proliferation and upregulates PD‐1 expression, suggesting a tumor‐promoting role for NFAT1 in HCC. However, these findings appear to conflict with those of Xu et al. [13], who proposed that NFAT inhibits tumor progression by enhancing expression of the FasL apoptotic pathway. Thus, the precise role of NFAT in HCC remains controversial, and the downstream pathways involved are not yet fully elucidated.

Mendelian randomization (MR) is a methodological approach widely used to infer causal relationships between exposures and outcomes. It typically employs genetic variants—most commonly single nucleotide polymorphisms (SNPs)—that are robustly associated with the exposure of interest as instrumental variables. Because genetic variants are randomly assorted at conception and inherited independently, they are generally less susceptible to confounding and reverse causation than traditional observational factors [14, 15]. MR thereby offers a valuable framework for estimating causal effects, often providing more reliable inferences than those derived from conventional observational studies.

In this context, we aim to identify key downstream genes associated with NFAT signaling by integrating genetic scores for NFAT, focusing on natural killer (NK) cells and T cells as the primary cell types of interest, and applying MR approaches. Our objective is to provide novel insights that may inform the diagnosis, management, and therapeutic targeting of HCC.

## 2. Results

### 2.1. Single‐Cell Transcriptome Reveals Five Major Cell Types and Identifies NK/T Cells as NFAT‐Active Populations

Following quality control, 80,673 cells and 21,584 genes from the GSE162616 dataset were retained for analysis (Figure S1). The Top 2000 highly variable genes were selected for downstream processing (Figure S2). Principal component analysis (PCA) was conducted, and the first 20 principal components were used for subsequent clustering (Figure S3). Uniform Manifold Approximation and Projection (UMAP) resolved the cells into 16 distinct clusters (Figure 1a, b), which were annotated as five cell types: T cells, B cells, hepatocytes, monocytes, and NK cells. NK cells constituted the most abundant population across all samples (Figures 1c, 1d, and 1e). High specificity of canonical marker genes supported the reliability of this annotation (Figure 1f). Gene set variation analysis (GSVA) revealed significant differences in metabolic pathways—such as aminoacyl‐tRNA biosynthesis, alanine/aspartate/glutamate metabolism, and ascorbate/aldarate metabolism—across the identified clusters (Figure 2a).

Figure 1Identification of differential cells. (a) UMAP clustering of all cells. (b) UMAP clustering stratified by HCC and normal groups. (c) Annotated cell types in all samples. (d) Annotated cell types in HCC and normal groups. (e) Proportions of cell types across samples. (f) Bubble heatmap of Top10 marker genes per cell type. UMAP, uniform manifold approximation and projection; HCC, hepatocellular carcinoma.

**Figure:**
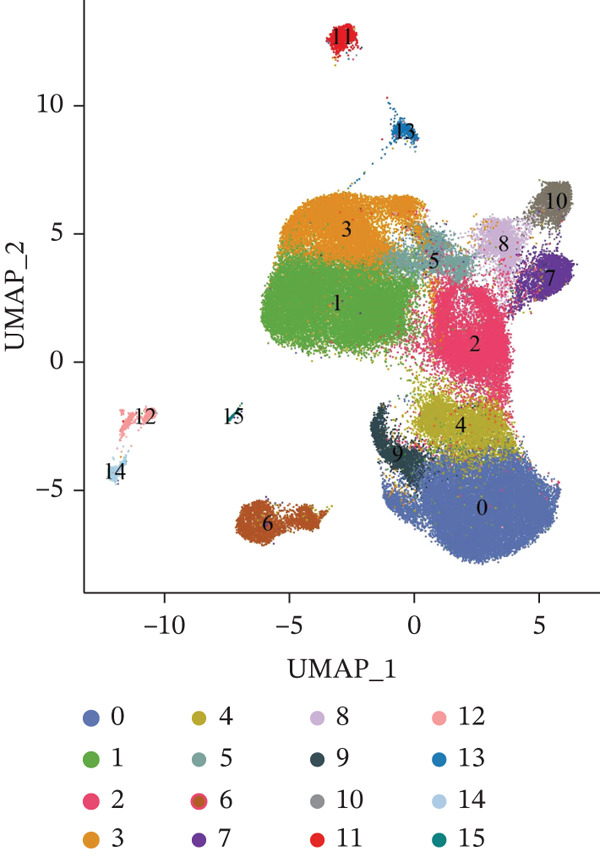
(a)

**Figure:**
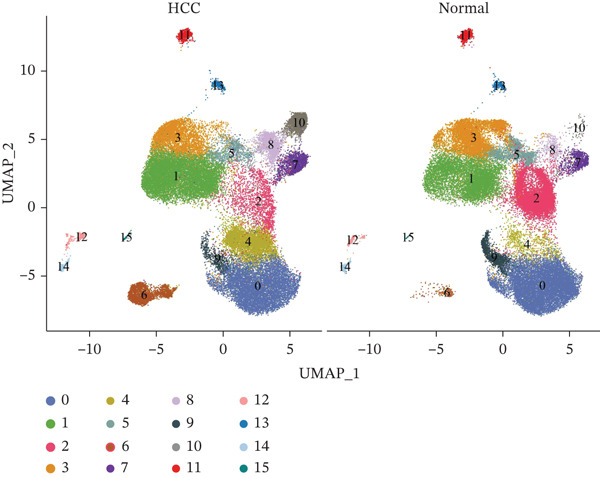
(b)

**Figure:**
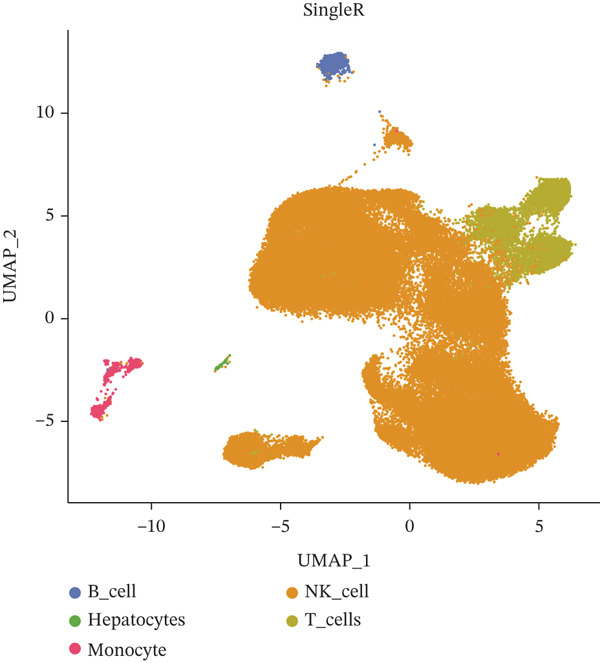
(c)

**Figure:**
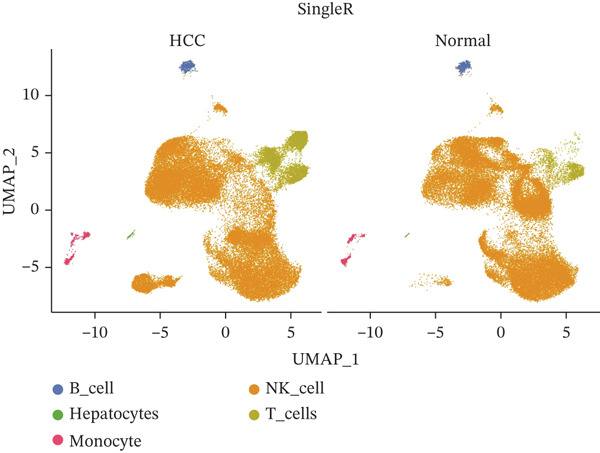
(d)

**Figure:**
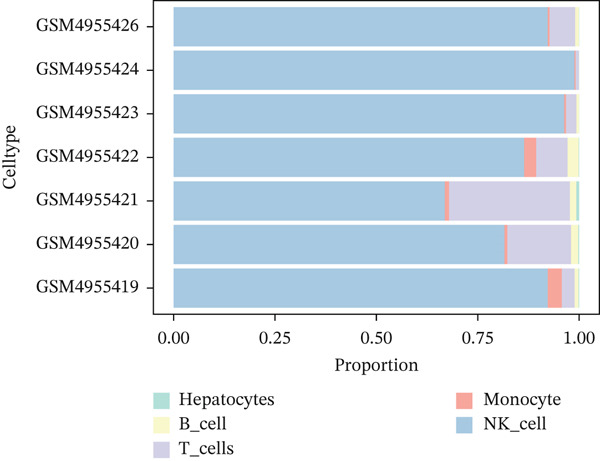
(e)

**Figure:**
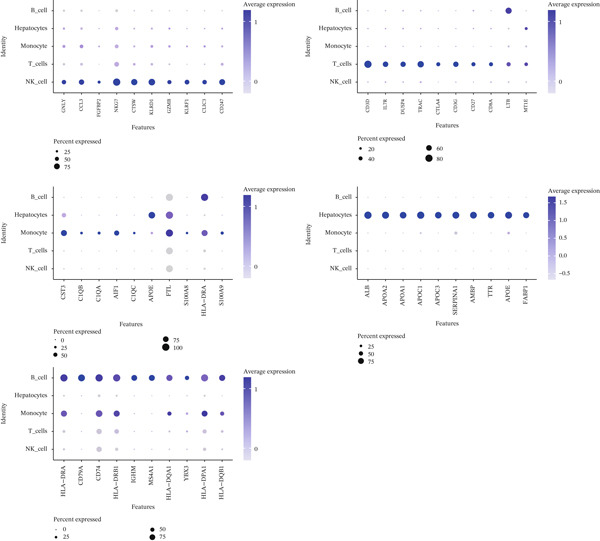
(f)

Figure 2GSVA‐based pathway activity and NFAT gene scores. (a) KEGG pathway enrichment across cell types. (b) NFAT activity scores in NK and T cells. KEGG, Kyoto Encyclopedia of Genes and Genomes.

**Figure:**
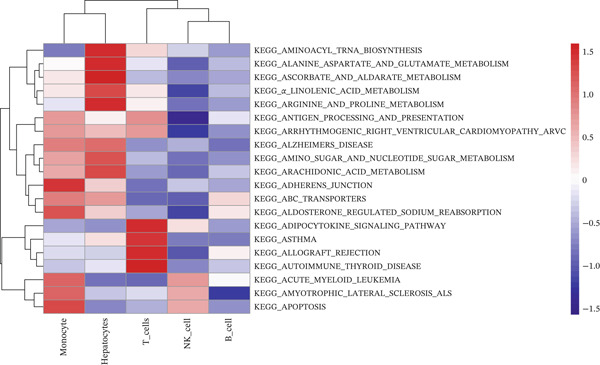
(a)

**Figure:**
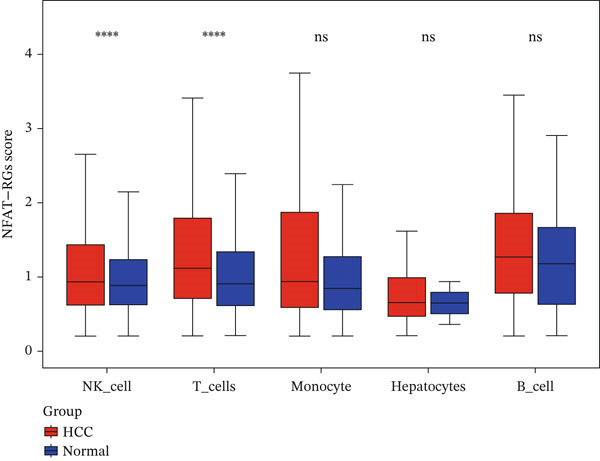
(b)

To investigate NFAT activity, we employed a *z*‐score algorithm to calculate pathway activity scores for each cell type. Notably, NK cells and T cells from HCC samples exhibited significantly higher NFAT activity scores compared with their counterparts in normal samples; these were therefore designated as “differential cells” for subsequent focused analysis (Figure 2b).

### 2.2. Differentially Expressed Nuclear Factor of Activated T Cells‐Related Genes (DE‐NFAT‐RGs) Are Predominantly Associated With Immune and Cytokine Functions

Differential expression analysis between HCC and normal samples identified 13 differentially expressed genes (DEGs) in NK cells and 40 in T cells. Combined, these constituted 46 unique DE‐NFAT‐RGs, including DUSP4, CTLA4, IGHG3, and IGKC (Figure 3a, b). Gene Ontology (GO) enrichment analysis revealed that these DE‐NFAT‐RGs were significantly enriched in immune‐related biological processes, such as regulation of T cell activation, lymphocyte‐mediated immunity, and positive regulation of leukocyte activation. Complementing this, Kyoto Encyclopedia of Genes and Genomes (KEGG) pathway analysis showed significant associations with cytokine–cytokine receptor interaction, autoimmune thyroid disease, and other immune‐related pathways (Figure 3c, d).

Figure 3DE‐NFAT‐RGs are enriched in immune and cytokine functions. (a) Differentially expressed genes in NK cells. (b) Differentially expressed genes in T cells. (c) GO enrichment of DE‐NFAT‐RGs. (d) KEGG enrichment of DE‐NFAT‐RGs. DE‐NFAT‐RGs, differentially expressed NFAT‐related genes; NK, natural killer; GO, gene ontology; NFAT‐RGs, nuclear factor of activated T‐cells related genes; KEGG, Kyoto Encyclopedia of Genes and Genomes.

**Figure:**
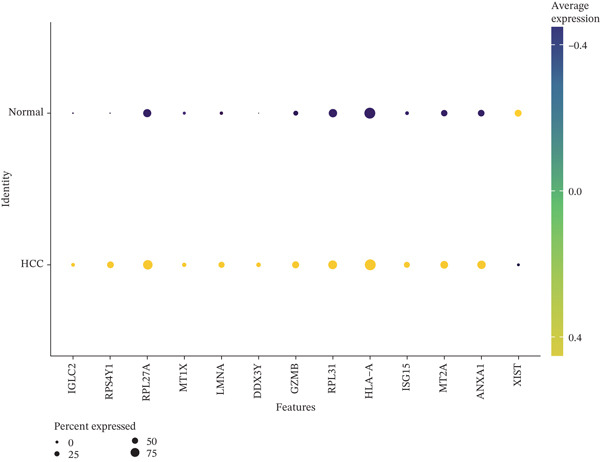
(a)

**Figure:**
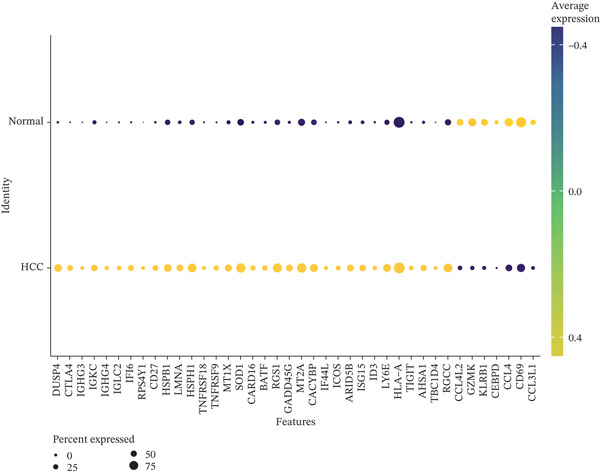
(b)

**Figure:**
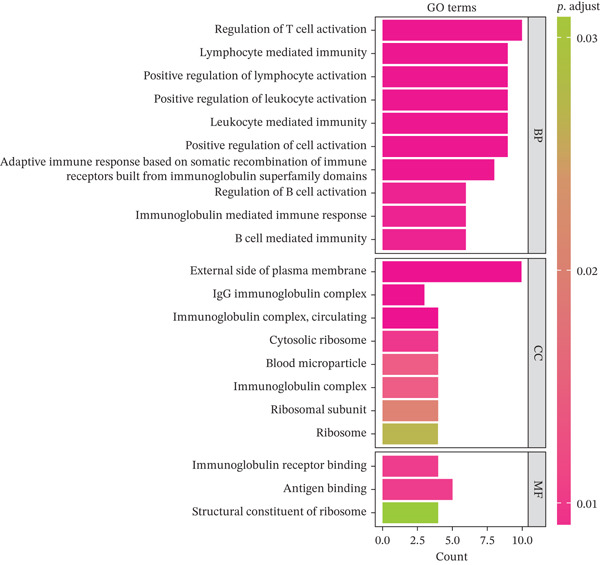
(c)

**Figure:**
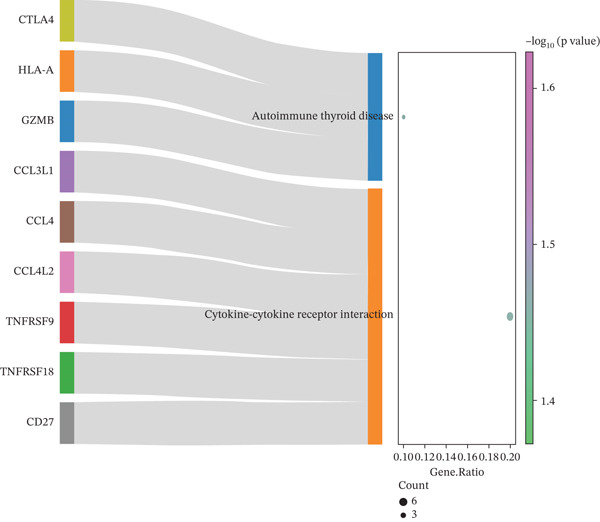
(d)

### 2.3. MR Identifies Calcyclin‐Binding Protein (CACYBP), CTLA4, and RGCC as Causal Hub Genes for HCC

We performed two‐sample MR on 29 DE‐NFAT‐RGs for which suitable expression quantitative trait loci (eQTL) instruments were available. This analysis identified three genes with significant causal associations with HCC. CTLA4 was associated with a decreased risk of HCC (odds ratio [OR] = 0.99967, 95% confidence interval [CI]: 0.99936–0.99998, *p* = 0.0363). Conversely, CACYBP (OR = 1.001, 95% CI: 1.0005–1.0013, *p* = 2.21*e* − 05) and RGCC (OR = 1.0003, 95% CI: 1.00008–1.00053, *p* = 0.0086) were associated with an increased risk (Figure 4a). Scatter, forest, and funnel plots confirmed the robustness of the MR estimates (Figures S4, S5, and S6). Sensitivity analyses revealed no significant heterogeneity (Cochran′s Q *p* > 0.05, Table 1) nor horizontal pleiotropy (MR‐Egger intercept *p* > 0.05, Table 2). Leave‐one‐out analysis confirmed that no SNP was driving the observed associations (Figure 4b). The Steiger directionality test further validated that the causal direction was from gene expression to HCC (correct_causal_direction = 1, steiger_test_adj < 0.05; Table S1). Based on these results, CACYBP, CTLA4, and RGCC were designated as hub genes for all subsequent analyses.

Figure 4Mendelian randomization identifies causal hub genes. (a) Forest plot of MR estimates for 29 DE‐NFAT‐RGs; CACYBP, CTLA4, and RGCC show significant causal associations with HCC. (b) Leave‐one‐out sensitivity analysis for the three hub genes. MR, Mendelian randomization; NFAT‐RGs, nuclear factor of activated T‐cells related genes.

**Figure:**
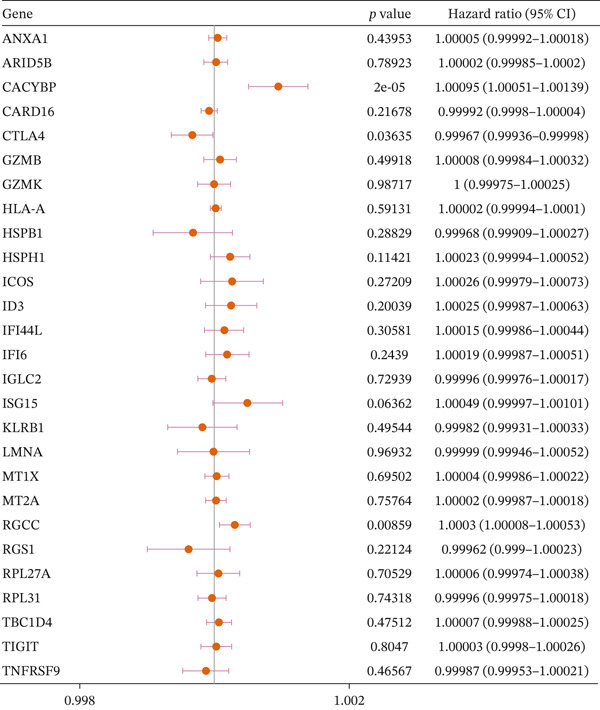
(a)

**Figure:**
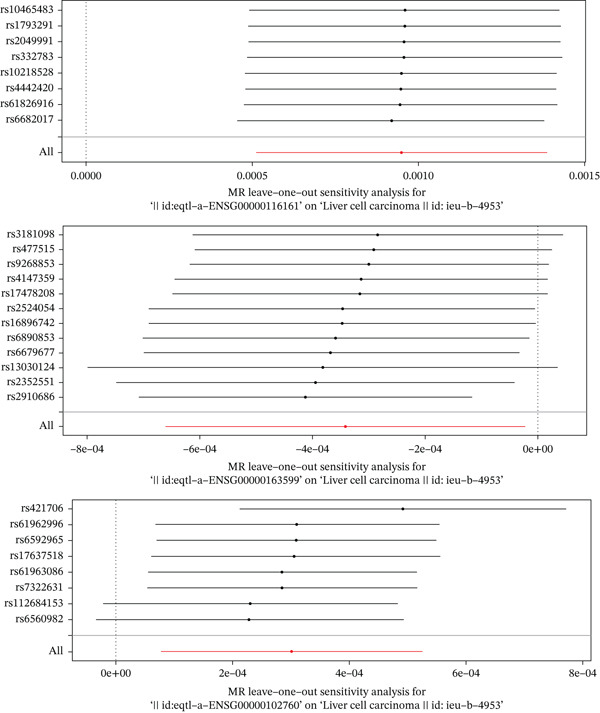
(b)

**Table 1:** Heterogeneity test for MR analysis of hub genes.

Gene	Method	*Q*	Q_df	Q_pval
RGCC	MR Egger	6.9184	6	0.328
	Inverse variance weighted	6.9870	7	0.430
CACYBP	MR Egger	0.0630	6	1.000
	Inverse variance weighted	0.1841	7	1.000
CTLA4	MR Egger	13.2618	10	0.209
	Inverse variance weighted	13.4935	11	0.262

**Table 2:** Horizontal pleiotropy test.

Gene	Egger_intercept	se	pval
CACYBP	0.00012	0.00035	0.7398
CTLA4	−0.00002	0.00005	0.6848
RGCC	−0.00001	0.00004	0.8155

### 2.4. T Cells and NK Cells Are the Key Cellular Compartments for Hub Gene Action

The expression distributions of the three hub genes were visualized within the differential cell populations (Figure 5a, b). Cell types in which all three hub genes were significantly differentially expressed between HCC and normal samples were defined as “key cells.” Both T cells and NK cells met this criterion. To further characterize these key populations, metabolic pathway activity scores were calculated using the AUCell algorithm. This analysis revealed that glycolysis/gluconeogenesis and oxidative phosphorylation were among the top five differentially activated pathways in both T cells and NK cells when comparing HCC with normal samples (Figure 5c, d).

Figure 5Identification of key cells. (a, b) Expression distribution of hub genes in NK cells (a) and T cells (b). (c, d) Top 5 differential metabolic pathways in NK cells (c) and T cells (d) between HCC and normal samples.

**Figure:**
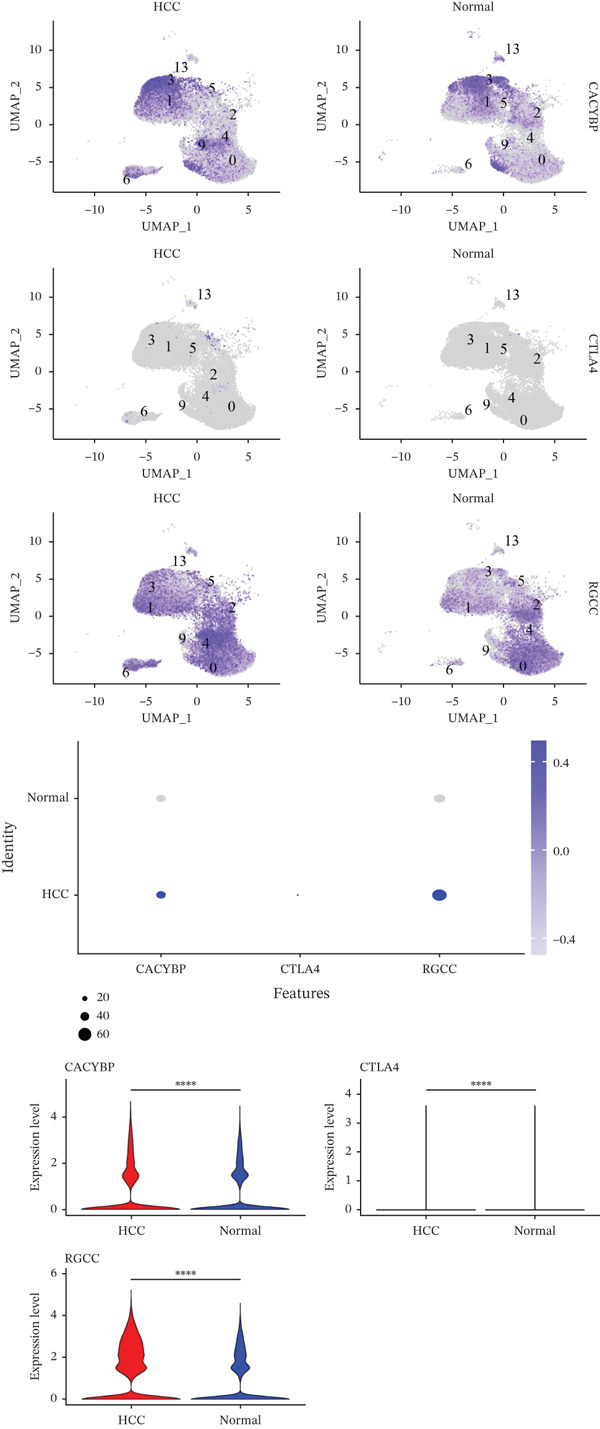
(a)

**Figure:**
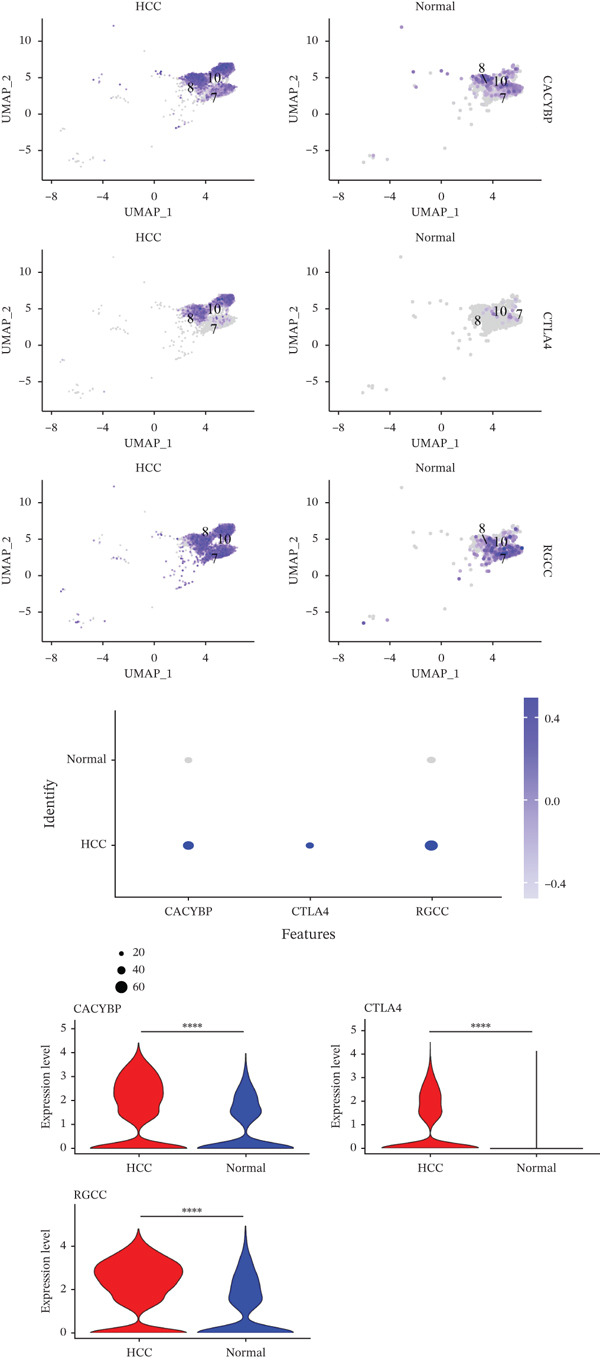
(b)

**Figure:**
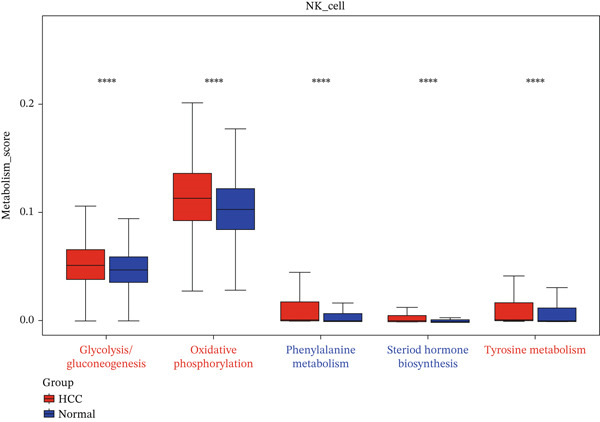
(c)

**Figure:**
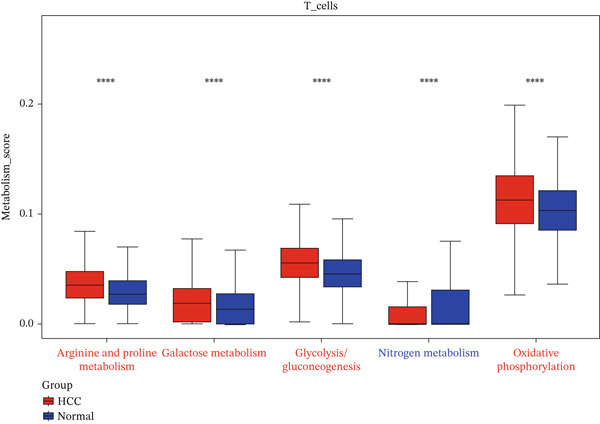
(d)

### 2.5. Pseudotime Trajectory Inference Reveals Dynamic Expression of Hub Genes During T Cell Differentiation

Pseudotime analysis of T cells was performed using Monocle3 to reconstruct their developmental trajectory. Cells were ordered along a continuum, starting from an early progenitor‐like state and progressing in maturity as pseudotime increased (Figures 6a, 6b, and 6c). The expression levels of CACYBP and CTLA4 changed significantly during differentiation, exhibiting an initial decrease followed by an increase. In contrast, RGCC expression remained stable initially before slowly declining (Figure 6d). This dynamic pattern suggests that CACYBP and CTLA4 may exert stage‐specific regulatory functions during T cell development, whereas RGCC may play a more constitutive role.

Figure 6Pseudotime trajectory of T cells. (a) Cells ordered along pseudotime (dark blue: early; light blue: late). (b) Nine differentiation states. (c) Distribution of T‐cell subclusters across pseudotime. (d) Dynamic expression of hub genes along pseudotime. CACYBP and CTLA4 initially decrease then increase; RGCC remains stable then slowly declines.

**Figure:**
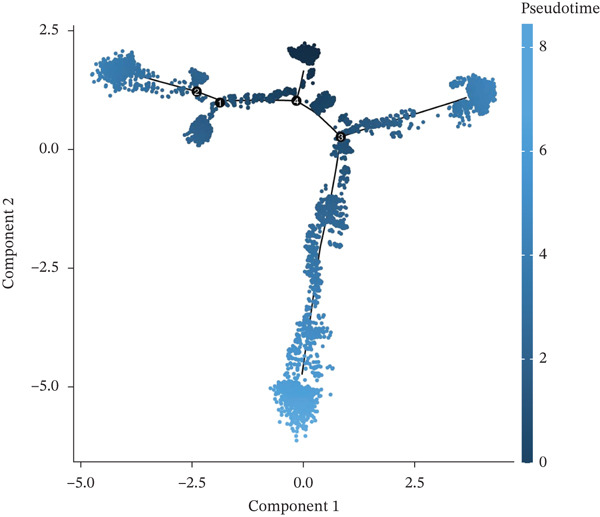
(a)

**Figure:**
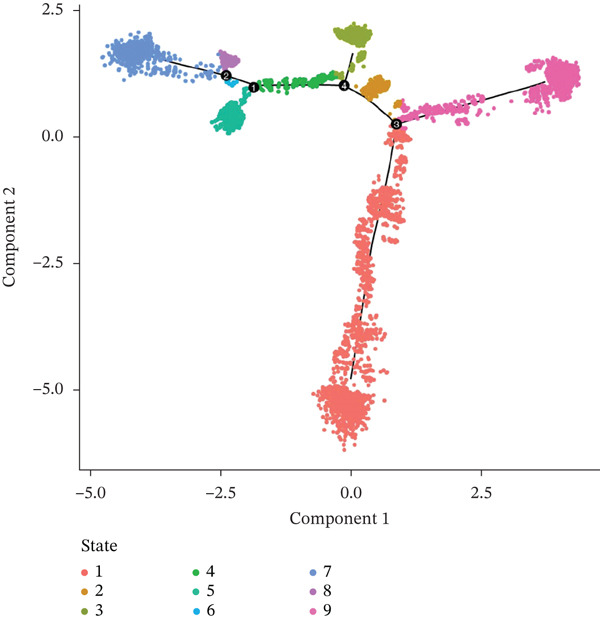
(b)

**Figure:**
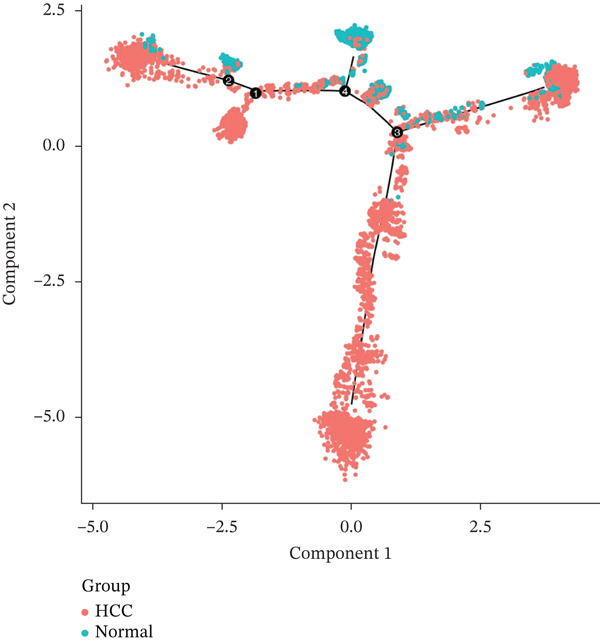
(c)

**Figure:**
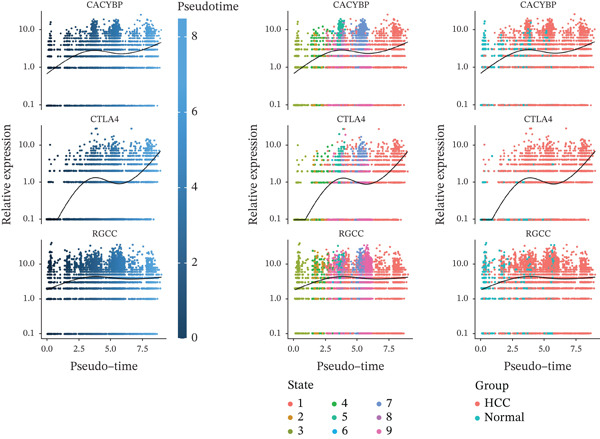
(d)

### 2.6. Cell–Cell Communication Analysis Highlights the MIF–(CD74 + CXCR4) Axis in HCC

Intercellular communication networks were reconstructed using CellChat. In HCC samples, frequent interactions were observed among T cells, between T cells and NK cells, between T cells and B cells, among NK cells, between NK cells and B cells, and between NK cells and monocytes. Notably, T–T and NK–B interactions were particularly intense (Figure 7a). In normal samples, NK–NK, NK–B, NK–T, and NK–monocyte interactions were predominant, with NK–NK and NK–B interactions showing the highest intensity (Figure 7b). The dominant outgoing signal from both T cells and NK cells to B cells in both conditions was mediated by the MIF ligand‐receptor pair (CD74 + CXCR4) (Figure 7c, d). These findings suggest that targeting T cells, NK cells, or the MIF signaling pathway may offer immunotherapeutic benefits in HCC.

Figure 7Cell–cell communication networks. (a, b) Circle plots showing intercellular interaction strength in HCC (a) and normal (b) samples. (c, d) Bubble plots of ligand–receptor pairs in HCC (c) and normal (d). The MIF‐(CD74+CXCR4) axis is the predominant signal from T cells/NK cells to B cells in both conditions.

**Figure:**
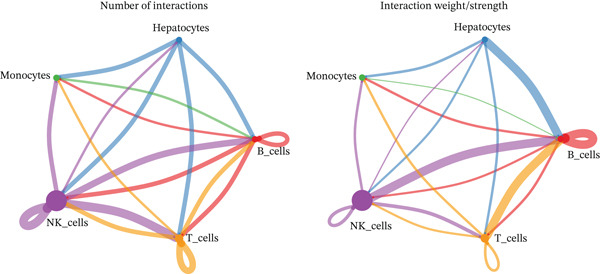
(a)

**Figure:**
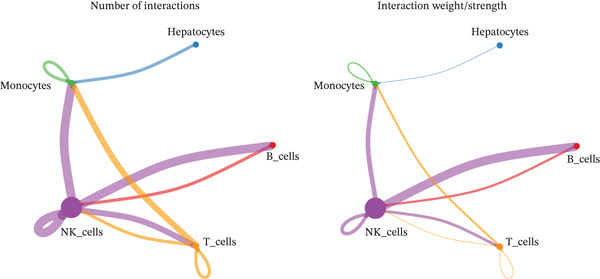
(b)

**Figure:**
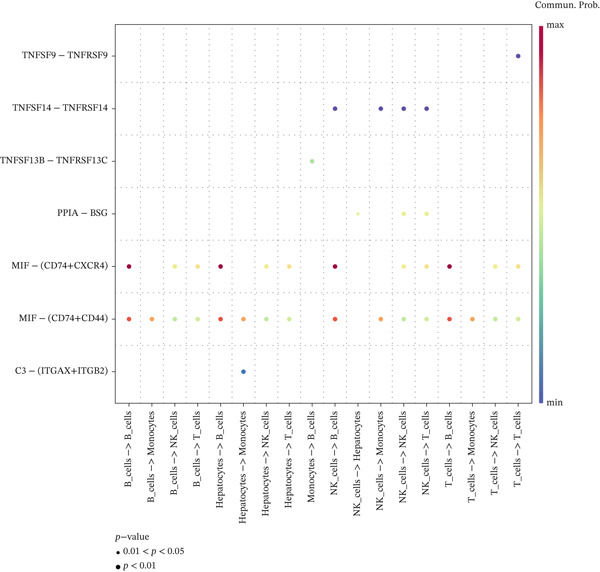
(c)

**Figure:**
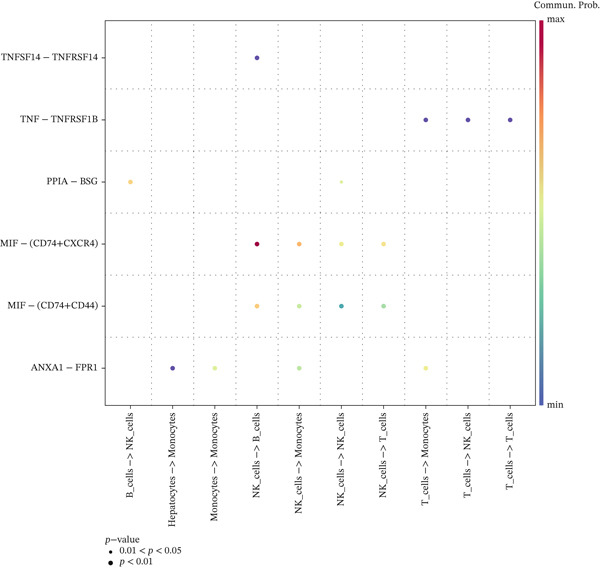
(d)

### 2.7. Elevated Expression of CACYBP, CTLA4, and RGCC in HCC and Their Clinical Relevance

Validation in The Cancer Genome Atlas Liver Hepatocellular Carcinoma (TCGA‐LIHC) cohort confirmed that CACYBP, CTLA4, and RGCC mRNA levels were significantly upregulated in HCC tissues compared with adjacent normal tissues (Figure 8a). Kaplan–Meier survival analysis revealed that high expression of CACYBP and RGCC was significantly associated with poorer OS, whereas CTLA4 expression did not show a significant prognostic effect (Figure 8b). Furthermore, CACYBP expression decreased with increasing patient age (Figure 8c). A nomogram integrating the three hub genes was constructed to predict patient outcomes (Figure 9a). The model′s performance was robust, with a calibration curve demonstrating strong agreement (mean absolute error < 0.1), a decision curve analysis (DCA) confirming clinical utility, and a receiver operating characteristic (ROC) curve yielding an area under the curve (AUC) of 0.9 (Figures 9b, 9c, and 9d).

Figure 8Validation of hub genes in TCGA‐LIHC. (a) Expression levels of CACYBP, CTLA4, and RGCC in HCC versus normal tissues. (b) Kaplan–Meier survival curves for high versus low expression of each hub gene. (c) Associations of hub gene expression with clinical parameters (age, grade, and stage).

**Figure:**
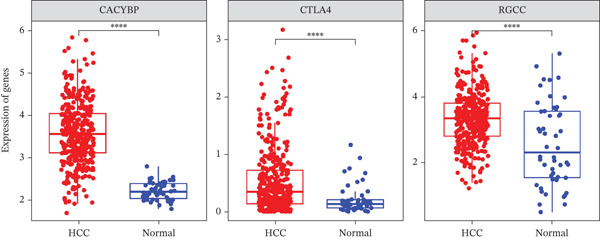
(a)

**Figure:**
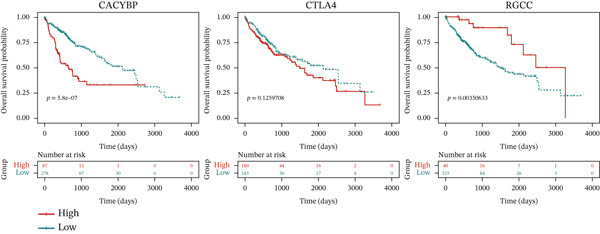
(b)

**Figure:**
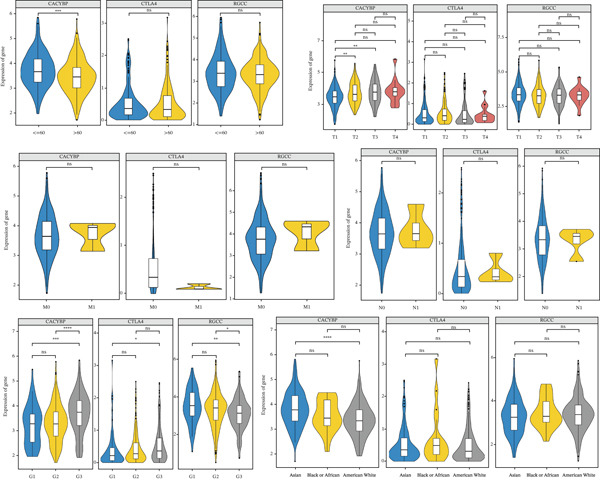
(c)

Figure 9Nomogram construction and evaluation. (a) Nomogram integrating the three hub genes. (b) Calibration curve. (c) Decision curve analysis. (d) ROC curve (AUC = 0.9).

**Figure:**
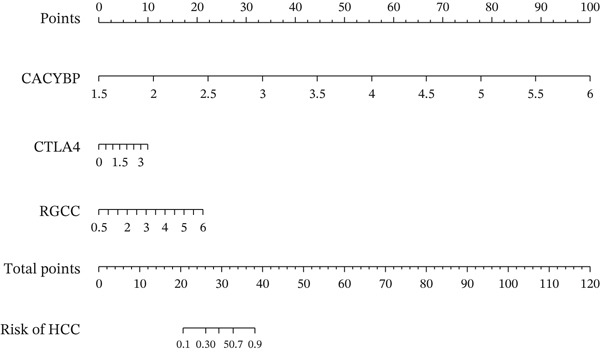
(a)

**Figure:**
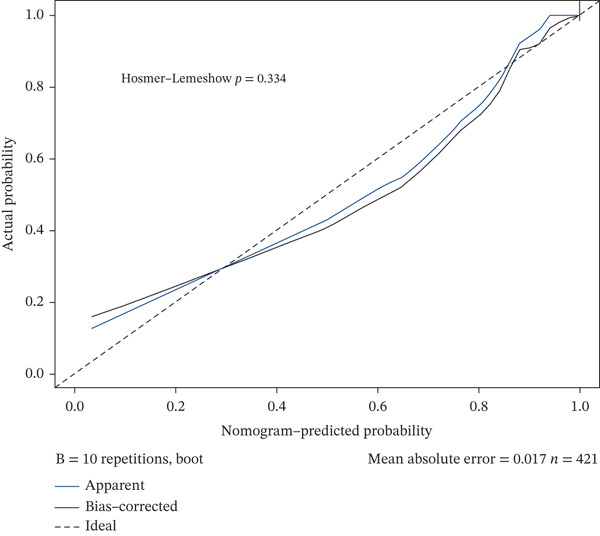
(b)

**Figure:**
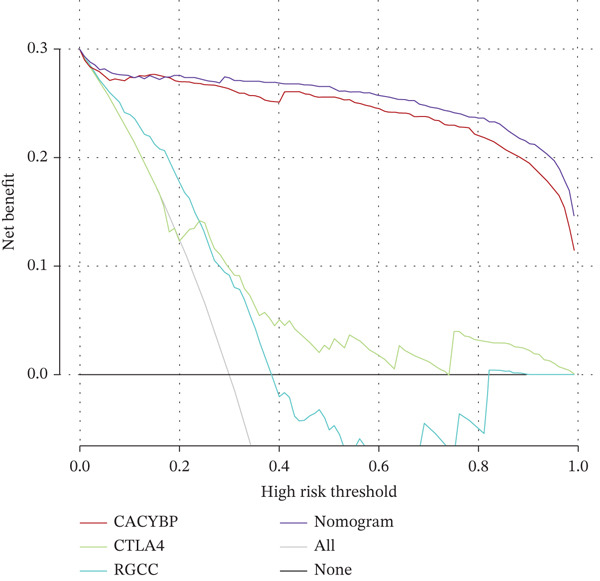
(c)

**Figure:**
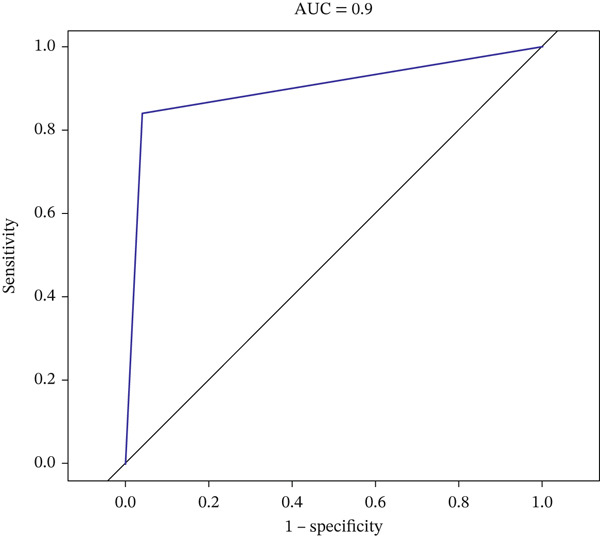
(d)

## 3. Materials and Methods

### 3.1. Data Sources

Gene expression matrices and corresponding clinical data for HCC were obtained from the University of California Santa Cruz (UCSC) Xena database (http://xena.ucsc.edu). Following log2 transformation, the dataset comprised 371 primary HCC tumor samples and 50 paired adjacent normal samples from TCGA‐LIHC cohort. A subset of 365 patients with complete survival information was extracted for subsequent survival analysis. The single‐cell RNA sequencing (scRNA‐seq) dataset GSE162616, consisting of four HCC and three normal samples, was downloaded from the Gene Expression Omnibus (GEO) (https://www.ncbi.nlm.nih.gov/gds). A core set of five canonical NFAT family genes—*NFATc1* (*NFAT2*), *NFATc2* (*NFAT1*), *NFATc3* (*NFAT4*), *NFATc4* (*NFAT3*), and *NFAT5*—was curated based on the existing literature [9].

### 3.2. scRNA‐Seq Analysis

Single‐cell data were processed using the Seurat package (v4.1.0) [16]. Quality control procedures excluded cells meeting any of the following criteria: fewer than 300 or more than 2000 detected genes, fewer than 6000 unique molecular identifiers (UMIs), or a mitochondrial read percentage exceeding 10%. After filtering, 80,673 cells were retained for downstream analysis. Data normalization and the identification of the Top 2000 highly variable genes were performed using the NormalizeData and FindVariableFeatures functions, respectively. PCA was conducted on the scaled data, and the first 20 principal components were selected based on JackStraw analysis. Cell clustering was performed using the UMAP method with a resolution parameter of 0.3. This specific resolution was chosen after evaluating a range from 0.1 to 0.5, as 0.3 yielded well‐separated clusters characterized by distinct marker gene expression and biologically meaningful cell types, consistent with standard practices in single‐cell studies [16]. Cell types were annotated using the HumanPrimaryCellAtlasData reference from the celldex package (v1.4.0) [17]. Marker genes for each cluster were identified using the FindAllMarkers function with parameters min.pct = 0.2 and only.pos = TRUE. GSVA was performed with the msigdbr package (v7.5.1) [18]using the c2.kegg.symbols.gmt background gene set.

### 3.3. Identification of DE‐NFAT‐RGs

Nuclear factor of activated T cells‐related gene (NFAT‐RG) activity scores were computed for each individual cell using the *z*‐score algorithm implemented in the GSVA package (v4.1.0) [19]. These scores were subsequently compared between HCC and normal samples within each cell type using the Wilcoxon test. Cell types exhibiting significantly different activity scores were designated as “differential cells.” DE‐NFAT‐RGs were identified using the FindMarkers function, applying thresholds of |log₂fold change (FC)| > 1, an adjusted *p* value < 0.05, and expression in at least 25% of cells within a given population. GO and KEGG enrichment analyses were carried out using the ClusterProfiler package (v4.2.2) [20] with an adjusted *p* value < 0.05 considered statistically significant.

### 3.4. MR Analysis

To identify DE‐NFAT‐RGs with a causal association with HCC, a two‐sample MR analysis was conducted. eQTL summary statistics for the DE‐NFAT‐RGs were obtained from the CAGE study, which includes 2765 individuals of European ancestry and uses peripheral blood samples [21]. Genome‐wide association study (GWAS) summary data for HCC (ieu‐b‐4953) were retrieved from the IEU OpenGWAS database (https://gwas.mrcieu.ac.uk), comprising 168 HCC cases and 372,016 controls (total *n* = 372,184). SNPs significantly associated with the exposure (gene expression) at a genome‐wide significance threshold (*p* < 5 × 10^−8^) were selected as potential instrumental variables. Linkage disequilibrium (LD) clumping was performed (*r* ^2^ = 0.001, clumping window = 10,000 kb) to ensure the independence of selected SNPs. The *F*‐statistic was calculated for each SNP, and only those with an *F*‐statistic > 10 were retained to ensure instrument strength and minimize the risk of weak instrument bias [15, 22]. The inverse variance weighted (IVW) method was employed as the primary MR analysis due to its provision of the most precise causal estimate under the assumption of no horizontal pleiotropy [23]. Several supplementary methods—MR‐Egger [24], weighted median [25], simple mode [22], and weighted mode [26]—were conducted as sensitivity analyses to assess the robustness of the findings. Heterogeneity among SNP estimates was evaluated using Cochran′s *Q* test [27], and horizontal pleiotropy was assessed using the MR‐Egger intercept test [28]. A leave‐one‐out analysis was performed to determine if any single SNP disproportionately influenced the causal estimate. Finally, the Steiger directionality test [29]was applied to exclude the possibility of reverse causation. Genes that passed the Steiger test (*p* < 0.05) were subsequently designated as hub genes for further investigation.

### 3.5. Key Cell Analysis

The expression patterns of the identified hub genes were visualized within the differential cell populations. Cells exhibiting significant differential expression of all three hub genes between HCC and normal samples were defined as “key cells.” To further characterize these populations, metabolic pathway activity in key cells was quantified using the AUCell method, as implemented in the scMetabolism package (v0.2.1) [30], and pathway scores were subsequently compared between HCC and normal samples.

### 3.6. Pseudotime Analysis

Pseudotime trajectories of T cells were reconstructed to model their developmental dynamics using the Monocle3 package (v2.22.0) [31]. Changes in gene expression as a function of pseudotime were visualized to explore differentiation‐related dynamics in the expression of the hub genes.

### 3.7. Cell–Cell Communication Analysis

Intercellular communication networks were inferred using the CellChat package (v1.4.0) [32]. Ligand–receptor pairs expressed in both HCC and normal samples were identified, and communication probabilities were calculated to construct global interaction networks. The most significant signaling axes were highlighted in the corresponding figures and legends.

### 3.8. Hub Gene Validation and Nomogram Construction

The expression levels of the hub genes were compared between HCC and normal tissues in the TCGA‐LIHC cohort using the Wilcoxon test. OS was analyzed using the Kaplan–Meier method with a log‐rank test, applying the optimal expression cutoff determined by the survminer package (v0.4.9) [33]. Associations between hub gene expression and clinical parameters, including age, pathologic T/N/M stage, and histologic grade, were also assessed. A nomogram integrating the three hub genes was constructed using the rms package (v6.5‐0) [34] to provide a quantitative tool for predicting patient outcomes. The predictive performance of the nomogram was evaluated using calibration curves, DCA, and ROC curves.

### 3.9. Statistical Analysis

All statistical analyses were performed using R software (v4.1.0). Group comparisons were conducted using the Wilcoxon test unless otherwise specified. A *p* value < 0.05 was considered statistically significant for all analyses.

## 4. Discussion

HCC is a highly lethal primary liver malignancy and continues to rank among the most prevalent cancers worldwide [35]. Although substantial efforts have been directed toward elucidating the molecular mechanisms underlying HCC initiation and progression, the precise role of NFAT transcription factors—critical regulators of proinflammatory cytokine expression during immune responses—remains incompletely characterized. The NFAT family comprises five members evolutionarily related to the REL‐NF‐*κ*B transcription factor family: NFAT1 (NFATc2/NFATp), NFAT2 (NFATc1/NFATc), NFAT3 (NFATc4), NFAT4 (NFATc3/NFATx), and NFAT5 (TonEBP) [36]. In the present study, we integrated single‐cell transcriptomics with MR to systematically investigate NFAT‐related genes in HCC. By focusing on NK cells and T cells as key differential populations, we identified three hub genes—CACYBP, CTLA4, and RGCC—that are causally associated with HCC risk.

T cell‐mediated immunity plays a pivotal role in suppressing HCC progression [37]. However, interactions between immune checkpoint ligands on tumor cells and their cognate receptors on T cells can induce T cell exhaustion, leading to immune evasion and accelerated tumor growth. Immune checkpoint inhibitors that disrupt these interactions have become first‐line therapies for HCC. NK cells constitute 25%–50% of hepatic lymphocytes and serve as a primary defense against microbial infections and tumor invasion [38]. The abundance of NK cells in peripheral blood or liver tissue has been positively correlated with survival outcomes in HCC patients [39]. Disruption of the NK cell activating receptor/ligand axis is a key mechanism driving HCC development and progression [40]. Strategies to enhance NK cell function—including adoptive cell transfer, cytokine activation (e.g., IL‐15), and antibodies that promote antibody‐dependent cellular cytotoxicity (ADCC)—are under active investigation to overcome tumor immune evasion and redirect NK cells toward tumors.

CACYBP is a modular protein involved in diverse cellular processes. Although highly expressed in tumor cells and neurons [41], its expression in normal human tissues is restricted, with highest levels in brain and heart and lowest in stomach, liver, and spleen [42]. In pancreatic cancer, CACYBP promotes tumor growth by downregulating P27 and Rb while upregulating CyclinE and CDK2 [43]. In colon cancer, gastrin stimulation induces CACYBP nuclear translocation, where it binds SKP1 and promotes P27 ubiquitination and degradation, thereby enhancing proliferation [44]. In gliomas, CACYBP participates in Siah1‐mediated cytoplasmic P27 degradation, reducing tumor cell migration and invasion [45]. In HCC, CACYBP is highly expressed and associated with poor prognosis. Both in vitro and in vivo studies have demonstrated that HCC cell growth requires CACYBP expression [46]. Mechanistically, in the absence of RNF41‐mediated degradation, CACYBP enhances cytoplasmic retention of P27Kip1, thereby promoting HCC progression [46]. Depletion of CACYBP disrupts tumor‐associated macrophage (TAM) infiltration and the immunosuppressive microenvironment, enhancing sensitivity to anti‐PD‐1 therapy [47]. Consistent with these findings, multiple prognostic models have identified CACYBP as an independent risk factor for HCC [48, 49]. Our MR analysis similarly identified CACYBP as a risk gene for HCC, with increased expression and genetic variants contributing to HCC occurrence. Notably, CACYBP expression decreased with advancing patient age, and high expression correlated with poorer OS. In pathological grading [50], G3 tumors exhibited significantly higher CACYBP expression than G1 and G2 tumors, further supporting its role as a risk‐associated gene in HCC progression.

CTLA‐4 (cytotoxic T‐lymphocyte‐associated protein 4) is a key negative regulator of immune responses, primarily expressed on activated T cells and regulatory T cells (Tregs), with low expression on naïve T cells. It suppresses antitumor immunity in solid tumors, including HCC [51], and numerous clinical trials targeting CTLA‐4 are ongoing. Our MR analysis identified CTLA4 as a protective factor for HCC (OR = 0.99967), suggesting that genetically determined higher CTLA4 expression may reduce HCC risk. This finding may initially appear counterintuitive given the established role of CTLA‐4 as an immune checkpoint and the therapeutic efficacy of CTLA‐4 blockade in cancer. However, several considerations may reconcile this apparent contradiction. First, MR estimates reflect the lifelong cumulative effect of genetically determined gene expression on disease risk, which may differ from the acute pharmacological effects of checkpoint inhibition in established tumors. Second, CTLA‐4 expression on Tregs is essential for maintaining immune homeostasis and preventing autoimmunity; thus, higher constitutive CTLA‐4 expression could theoretically reduce chronic inflammation—a known risk factor for HCC development. Third, the observed protective effect may reflect differences between expression levels and functional states, or context‐dependent roles of CTLA‐4 in early versus late stages of hepatocarcinogenesis. Notably, our clinical correlation analysis revealed that CTLA4 expression was significantly higher in G3 tumors compared with G1 tumors, suggesting that once HCC is established, CTLA‐4 may contribute to tumor progression by promoting immune evasion. These findings underscore the complexity of CTLA‐4 biology in HCC and highlight the importance of considering both genetic predisposition and tumor stage when evaluating immunotherapeutic strategies. Although survival analysis did not reveal a significant association between CTLA4 expression and HCC survival—possibly due to sample size limitations—our results provide genetic evidence supporting CTLA‐4 as a relevant target in HCC immunotherapy.

RGCC (regulator of cell cycle, also known as RGC‐32) is a cell cycle regulatory factor widely expressed in multiple human organs [52]. Its expression can be induced by complement, growth factors, cytokines, angiogenic factors, and hormones [53]. RGCC plays primary roles in cell migration, differentiation, and fibrosis [52], and promotes aortic smooth muscle cell entry into S phase by directly binding to CDK1 and enhancing its kinase activity [54]. RGCC exerts protective effects in pulmonary fibrosis, where reduced expression leads to collagen deposition [55]. In triple‐negative breast cancer (TNBC), RGCC drives lung‐specific metastasis through the RGCC/PLK1/AMPK*α*2 axis, representing a potential therapeutic target [56]. As a cell cycle regulator, RGCC has been implicated in the development and invasiveness of various tumors, including colon cancer, ovarian cancer, cutaneous T‐cell lymphoma, and Burkitt′s lymphoma [52]. Recently, RGCC was included in a nine‐gene genomic‐clinicopathologic model for predicting mortality in HCC, suggesting its potential utility in personalized treatment [57]. However, reports on the correlation between RGCC and HCC biology remain limited.

Our study demonstrates that RGCC expression is significantly upregulated in HCC tissue compared with normal tissue, and high RGCC expression is associated with poorer OS. Furthermore, RGCC expression increased with advancing pathological grade, suggesting its involvement in HCC progression. Interestingly, MR analysis identified RGCC as a risk gene for HCC occurrence (OR = 1.0003), consistent with its protumorigenic role. However, the relationship between RGCC expression and survival may be more complex. Several factors could explain this complexity: (1) RGCC may exert dual mechanisms in tumors—although highly expressed, it might partially inhibit tumor growth or metastasis through context‐dependent effects on cell proliferation and apoptosis; (2) other genetic or molecular pathways, as well as treatment sensitivity, may counteract the negative impact of high RGCC expression; (3) as noted by Vlaicu et al., RGCC appears to play pleiotropic roles in different malignant contexts depending on cell line age, underlying ligands, and protein levels, determining whether cells accelerate proliferation or undergo mitotic arrest [52]. Thus, although RGCC clearly influences HCC development, its precise mechanisms warrant further investigation.

The tumor microenvironment (TME) comprises diverse cellular and acellular components that collectively drive tumor growth, invasion, metastasis, and therapeutic response [58]. In HCC, the TME exerts immunosuppressive effects, inducing tolerance and promoting tumor proliferation, invasion, and metastasis. This tumor‐promoting response is mediated by environmental factors, oncogenic processes, and a network of immunosuppressive cell subsets, inflammatory molecules, and signaling pathways [59]. Within this intricate signaling network, both the NK cells and T cells responsible for immune surveillance in early tumor development, and the Treg, CD8^+^, and CD4^+^ T cells that play critical roles during tumor progression, belong to the NK or T cell lineages. This underscores that NK cells, T cells, and their regulatory factors occupy central positions throughout HCC development. Accordingly, we focused on NK cells and T cells and their regulatory factors (NFAT), employing MR to mitigate confounding and more precisely identify immune‐related pathways influencing HCC pathogenesis. Our findings suggest that the identified hub genes may serve as key executors of immune regulation within the TME.

Based on the three hub genes, we constructed a nomogram prediction model and scoring system with HCC occurrence as the outcome variable. Among these, CACYBP contributed the largest proportion to the predicted probability of HCC, indicating that its expression level plays a dominant role in this model, whereas RGCC and CTLA4 exhibited some variability. This model, utilizing only three gene expression values as independent variables, offers a relatively simple and cost‐effective approach compared with multigene panels and holds promise for clinical prediction of HCC risk and evaluation of treatment response. A schematic diagram summarizing the key findings and proposed mechanisms of this study is presented in Figure S7.

## 5. Limitations and Future Directions

Several limitations of this study should be acknowledged. First, the MR analysis was based on eQTL data derived from peripheral blood of individuals of European ancestry, which may not fully capture tissue‐specific regulatory mechanisms or the genetic diversity of other populations. Second, the single‐cell dataset comprised a limited number of samples, and the findings require validation in larger, independent cohorts. Third, although we have established causal genetic links, the precise molecular mechanisms by which these hub genes influence T‐cell and NK‐cell functions within the HCC microenvironment remain to be experimentally validated. Future studies should employ in vitro coculture systems and in vivo HCC models to manipulate CACYBP, CTLA4, and RGCC expression in specific immune subsets and assess the consequent impact on tumor progression and immunotherapy response. Furthermore, prospective clinical studies are warranted to evaluate the predictive value of the derived nomogram in guiding early diagnosis and treatment decisions.

## 6. Conclusions

Collectively, by integrating scRNA‐seq with MR, this study identifies CACYBP, CTLA4, and RGCC as hub genes with causal roles in HCC progression. Our findings highlight T cells and NK cells as the principal cellular compartments through which these genes may exert their effects. The resultant three–gene‐based nomogram exhibits robust diagnostic accuracy and represents a valuable tool with significant potential for clinical translation, particularly in guiding early detection and immunotherapy stratification. Further investigation is warranted to delineate the precise molecular mechanisms underlying these associations and to prospectively validate the nomogram′s performance in multicenter cohorts.

## Author Contributions

Yunfei Chen, Xiting Yang, and Lintao Zhong have contributed to the work equally.

## Funding

The study was supported by the Project of Science & Technology Department of Sichuan Province key R&D plan (Grant Number: 23ZDYF2443) and the Research Project Plan of Sichuan Medical Association (Grant Number: S210018).

## Disclosure

All authors read and approved the final manuscript.

## Conflicts of Interest

The authors declare no conflicts of interest.

## General Statement

*Experimental Model and Subject Details*. This study is a computational analysis based on publicly available genomic and transcriptomic datasets. No cell lines, primary cells, or animal models were used in the experiments described herein. Therefore, requirements regarding cell line authentication, mycoplasma testing, and related descriptors are not applicable.

## Supporting Information

Additional supporting information can be found online in the Supporting Information section.

## Supporting information


**Supporting Information 1** Figure S1: Quality control metrics of single‐cell RNA sequencing data before and after filtering.

**Supporting Information 2** Figure S2: Selection of Top 2000 highly variable genes for downstream analysis.

**Supporting Information 3** Figure S3: Principal component analysis (PCA) scree plot and selection of the first 20 principal components.

**Supporting Information 4** Figure S4: Scatter plots illustrating the causal associations of *CACYBP*, *CTLA4*, and *RGCC* with hepatocellular carcinoma (HCC) in Mendelian randomization analysis.

**Supporting Information 5** Figure S5: Forest plots showing the effect estimates of instrumental variables for *CACYBP*, *CTLA4*, and *RGCC* on HCC risk using the inverse variance weighted (IVW) method.

**Supporting Information 6** Figure S6: Funnel plots assessing the symmetry and potential pleiotropy of genetic variants used in Mendelian randomization analysis.

**Supporting Information 7** Figure S7: Mechanism schematic diagram.

**Supporting Information 8** Table S1: Results of the Steiger directional test for reverse causation.

**Supporting Information 9** STROBE‐MR Checklist: A completed STROBE‐MR checklist is provided.

## Data Availability

The data that support the findings of this study are openly available in (UCSC) at (http://xena.ucsc.edu/), (GEO) at (https://www.ncbi.nlm.nih.gov/gds) Reference Number (GSE162616) and (GWAS) at (https://gwas.mrcieu.ac.uk/) Reference Number (ieu‐b‐4953).
