# Supplementary figures and images for: Single‐Cell Sequencing and Mendelian Randomization Reveal T Cell Nuclear Factor Genes in Hepatocellular Carcinoma Progression
Source: Hum Mutat. 2026 Apr 20;2026:7446280. doi: 10.1155/humu/7446280 (PMC13096692; doi:10.1155/humu/7446280)

**(a)**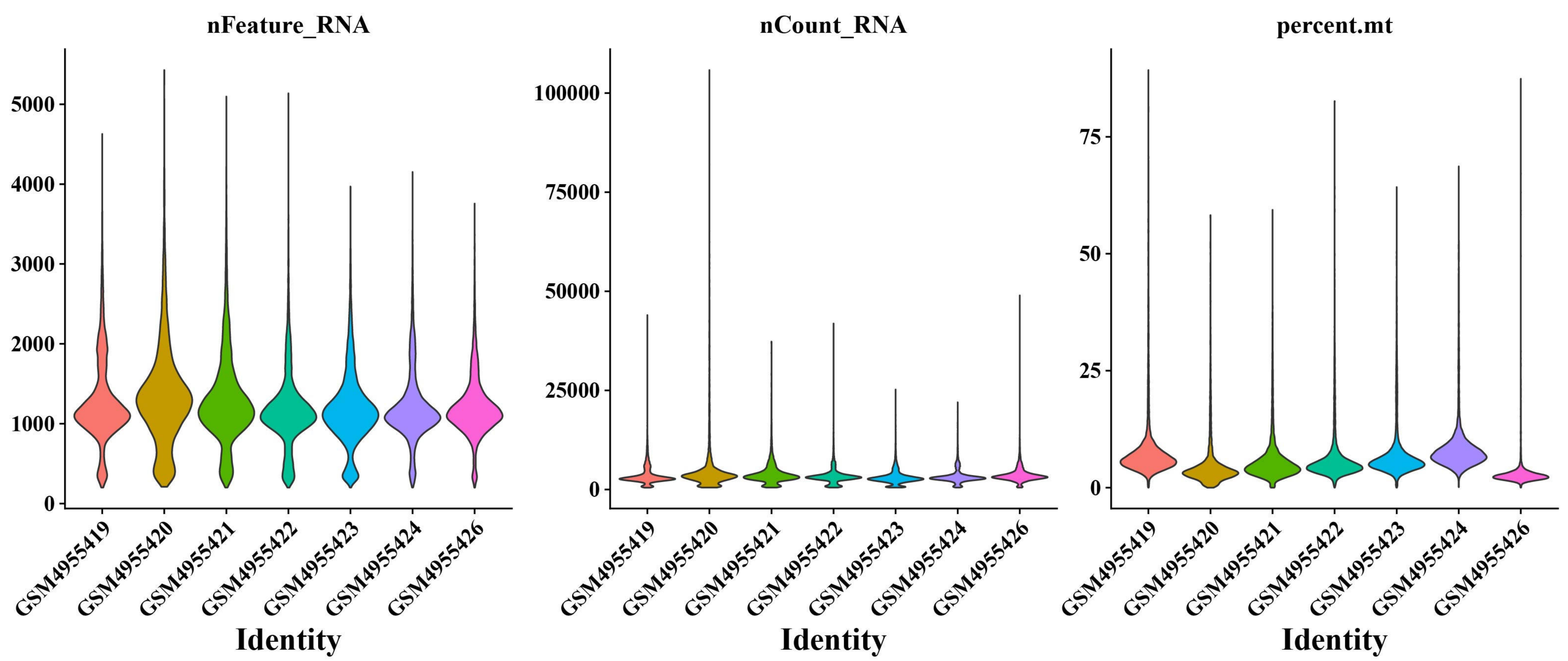**(b)**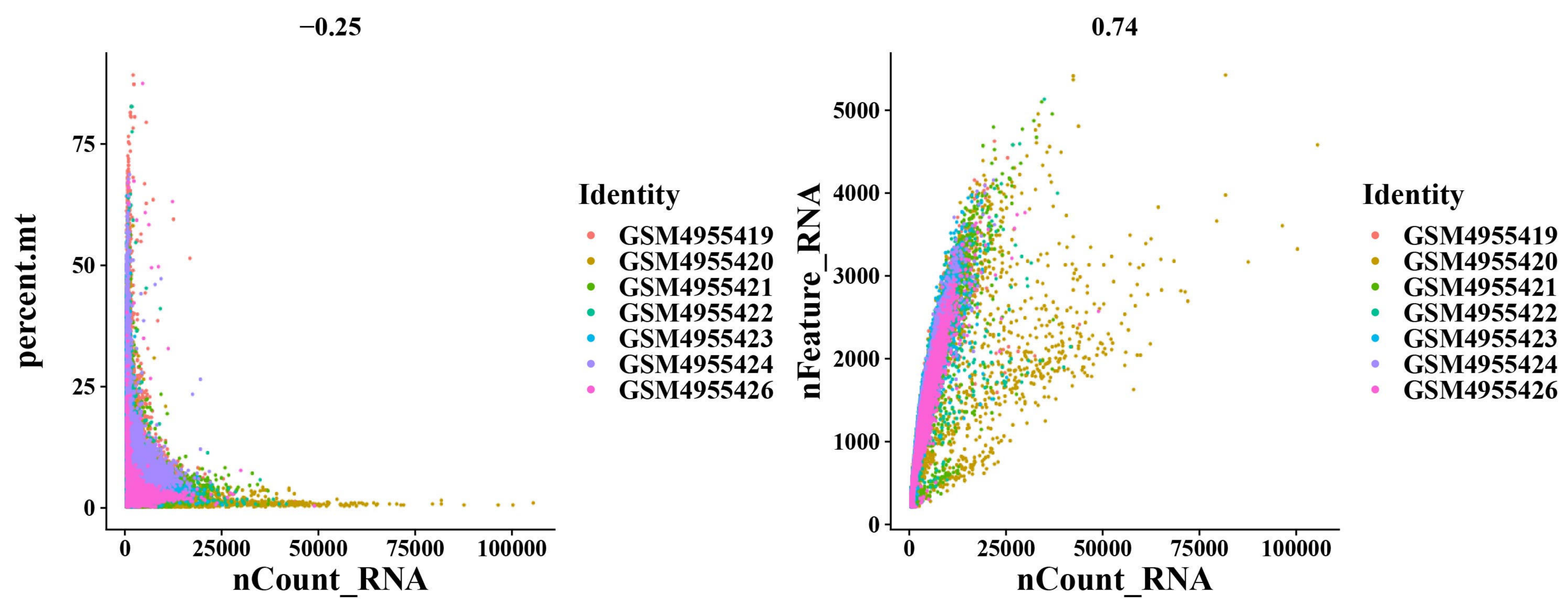**(c)**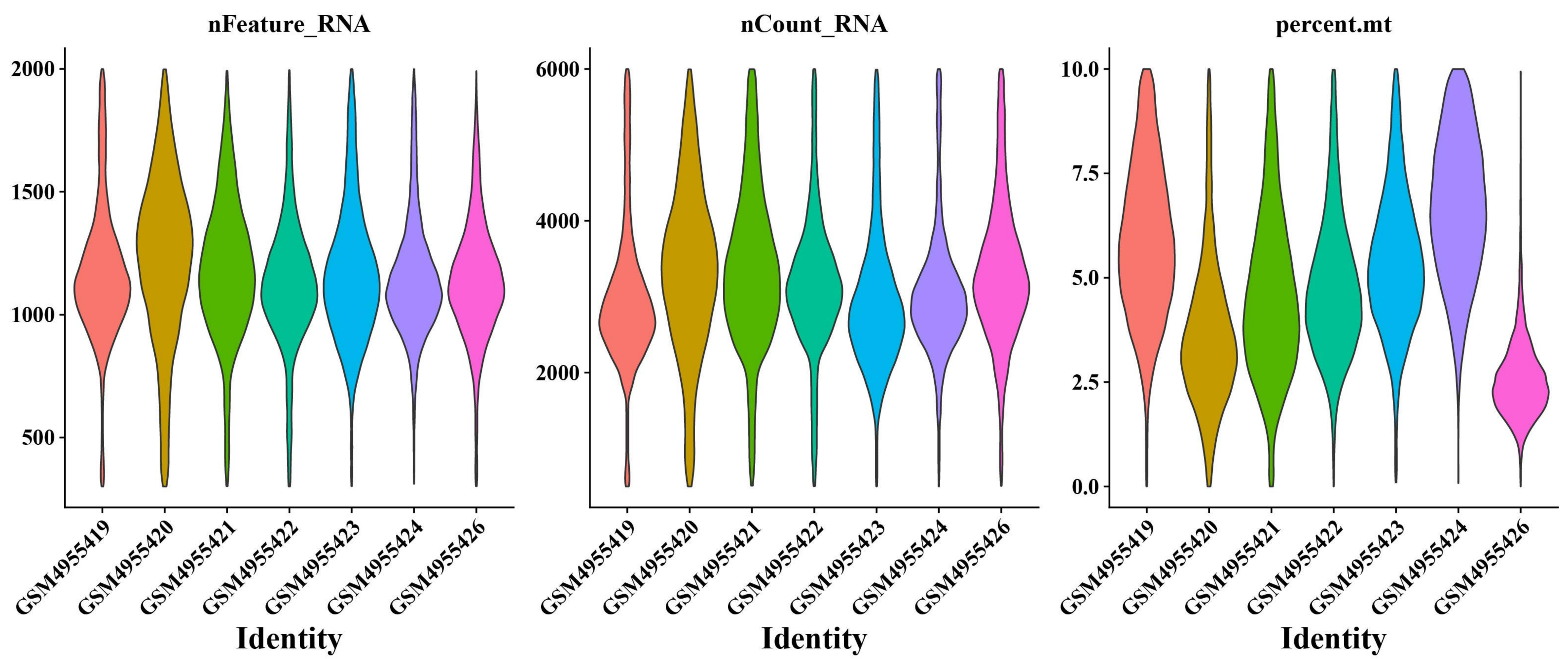**(d)**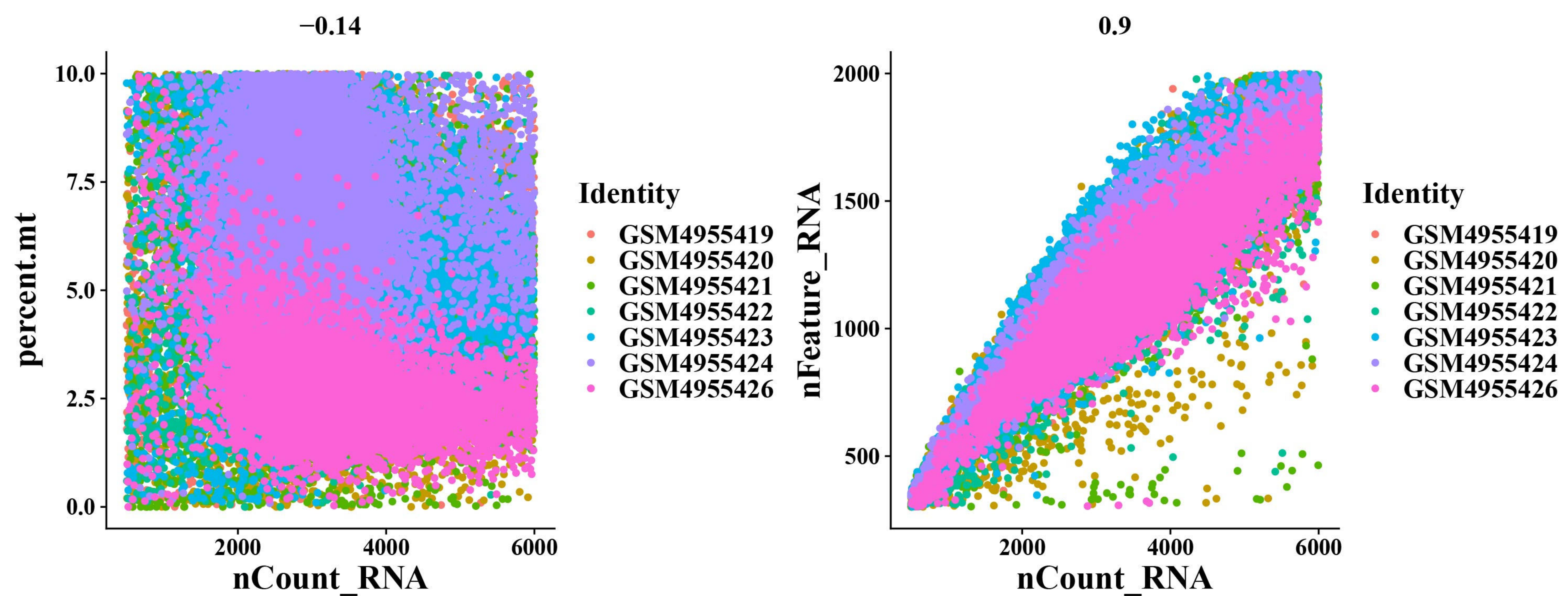

Supplement: Supplementary file 1 — Supporting Information 1 Figure S1: Quality control metrics of single‐cell RNA sequencing data before and after filtering. [file HUMU-2026-7446280-s001.pdf]

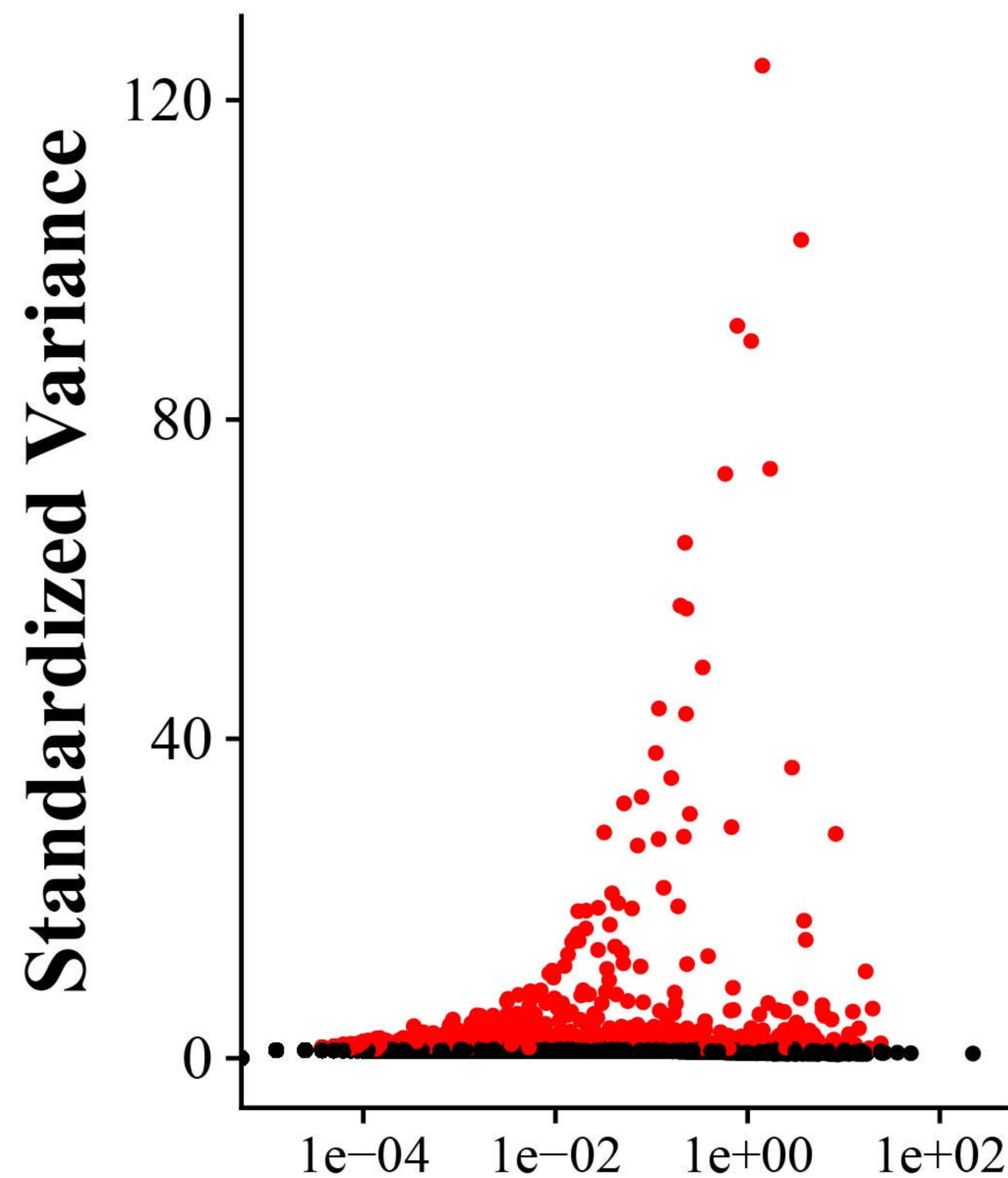

- Non-variable count: 19584
- Variable count: 2000

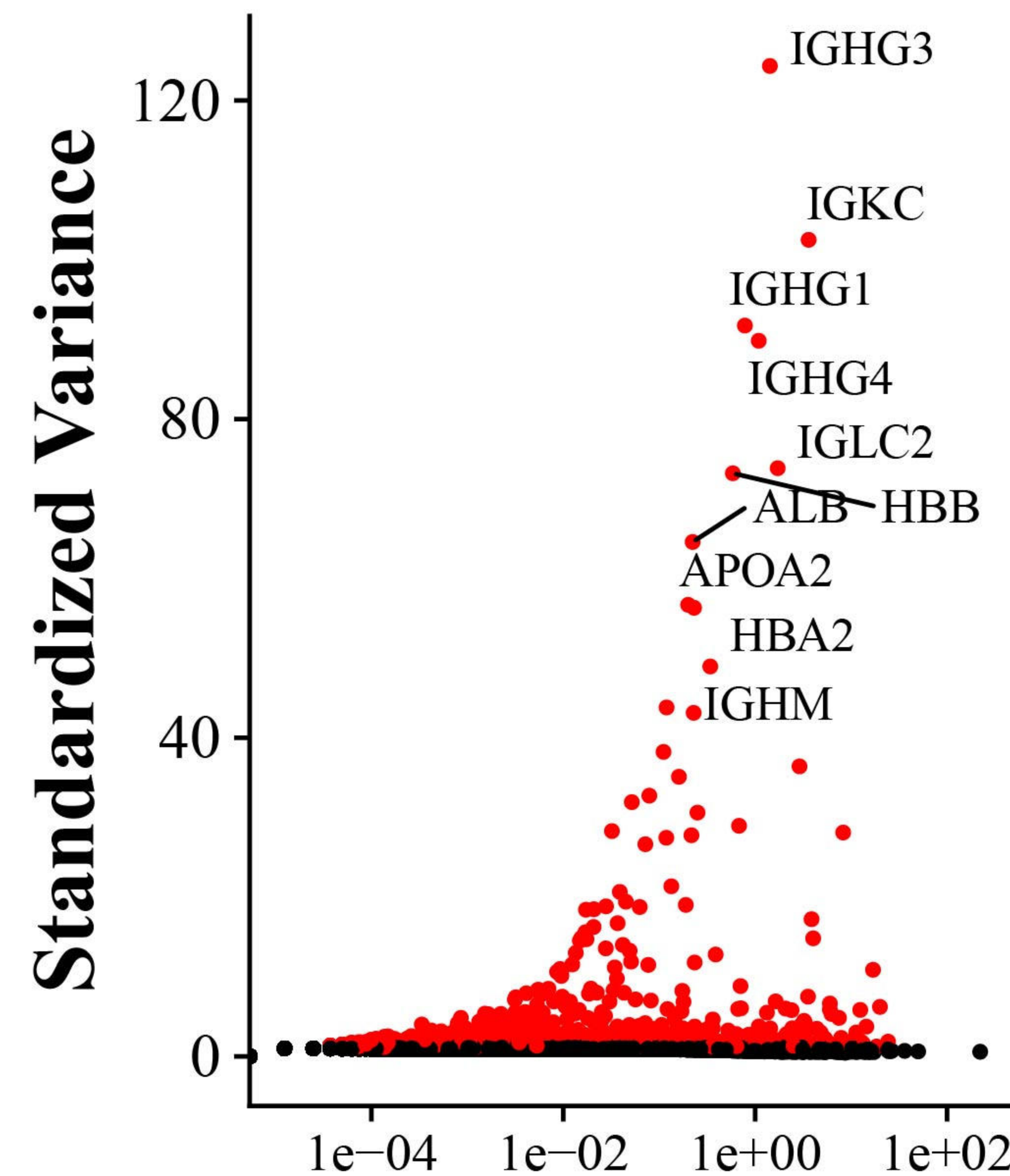

- Non-variable count: 19584
- Variable count: 2000

Supplement: Supplementary file 2 — Supporting Information 2 Figure S2: Selection of Top 2000 highly variable genes for downstream analysis. [file HUMU-2026-7446280-s002.pdf]

**(a)**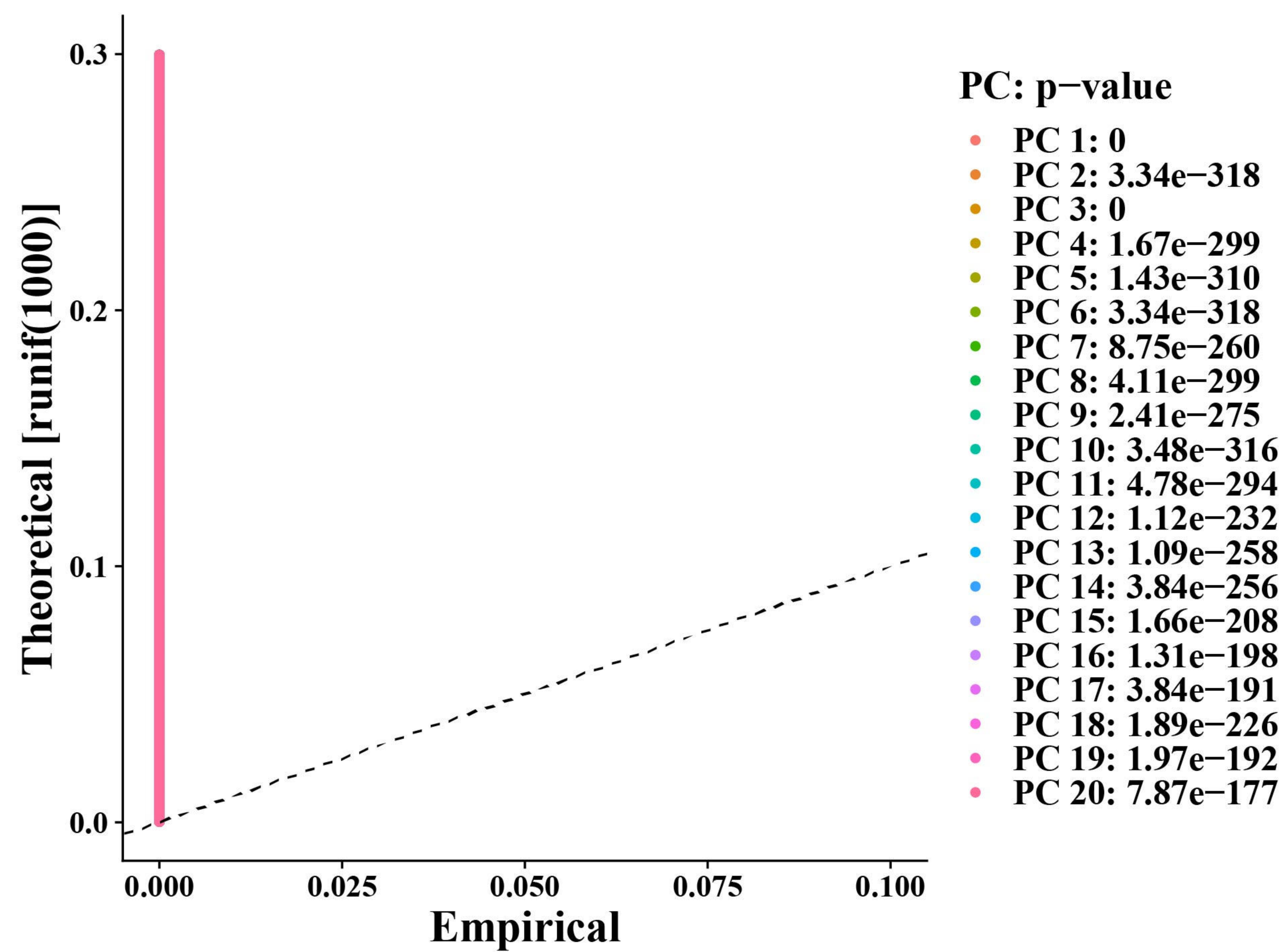**(b)**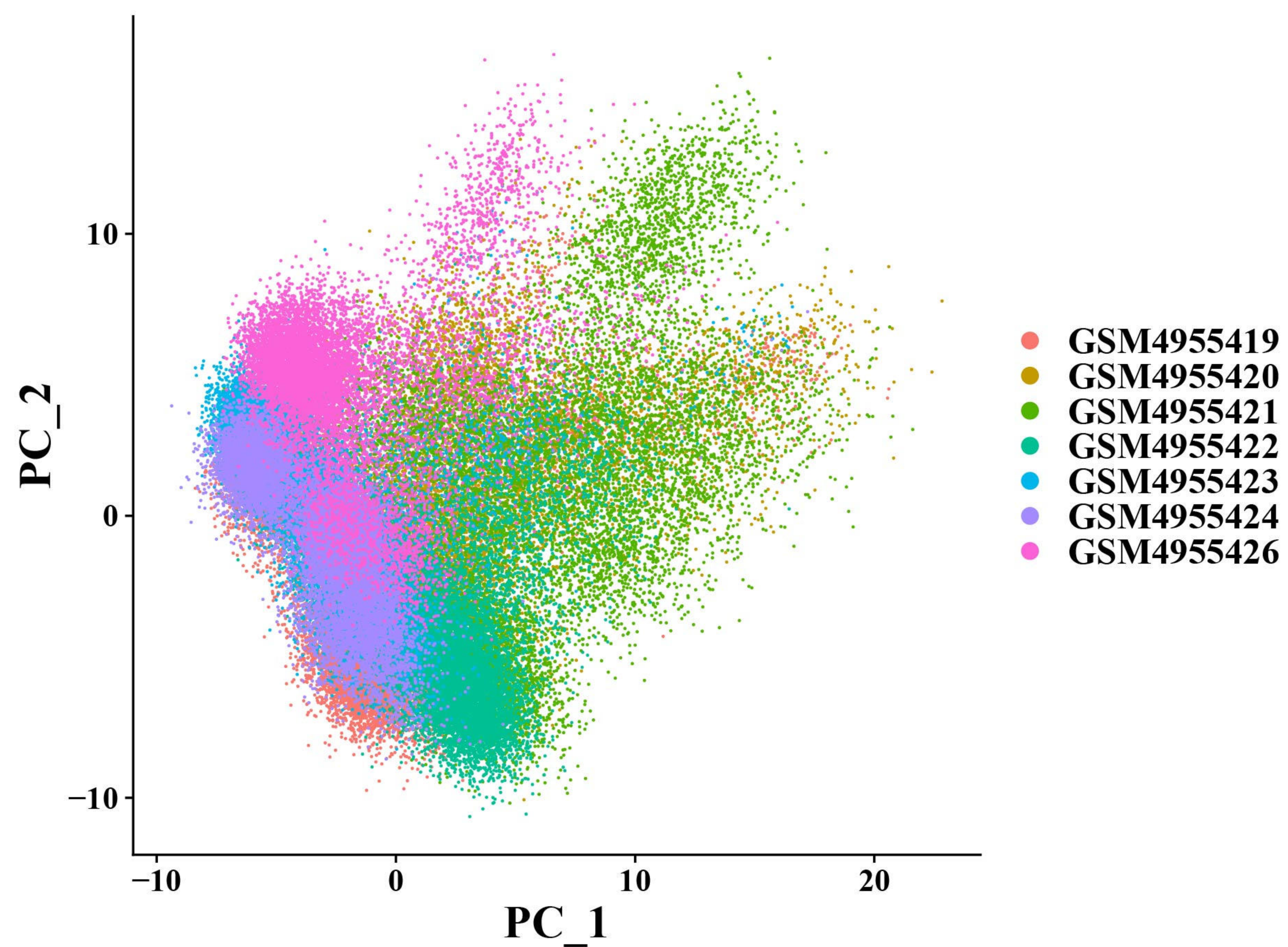**(c)**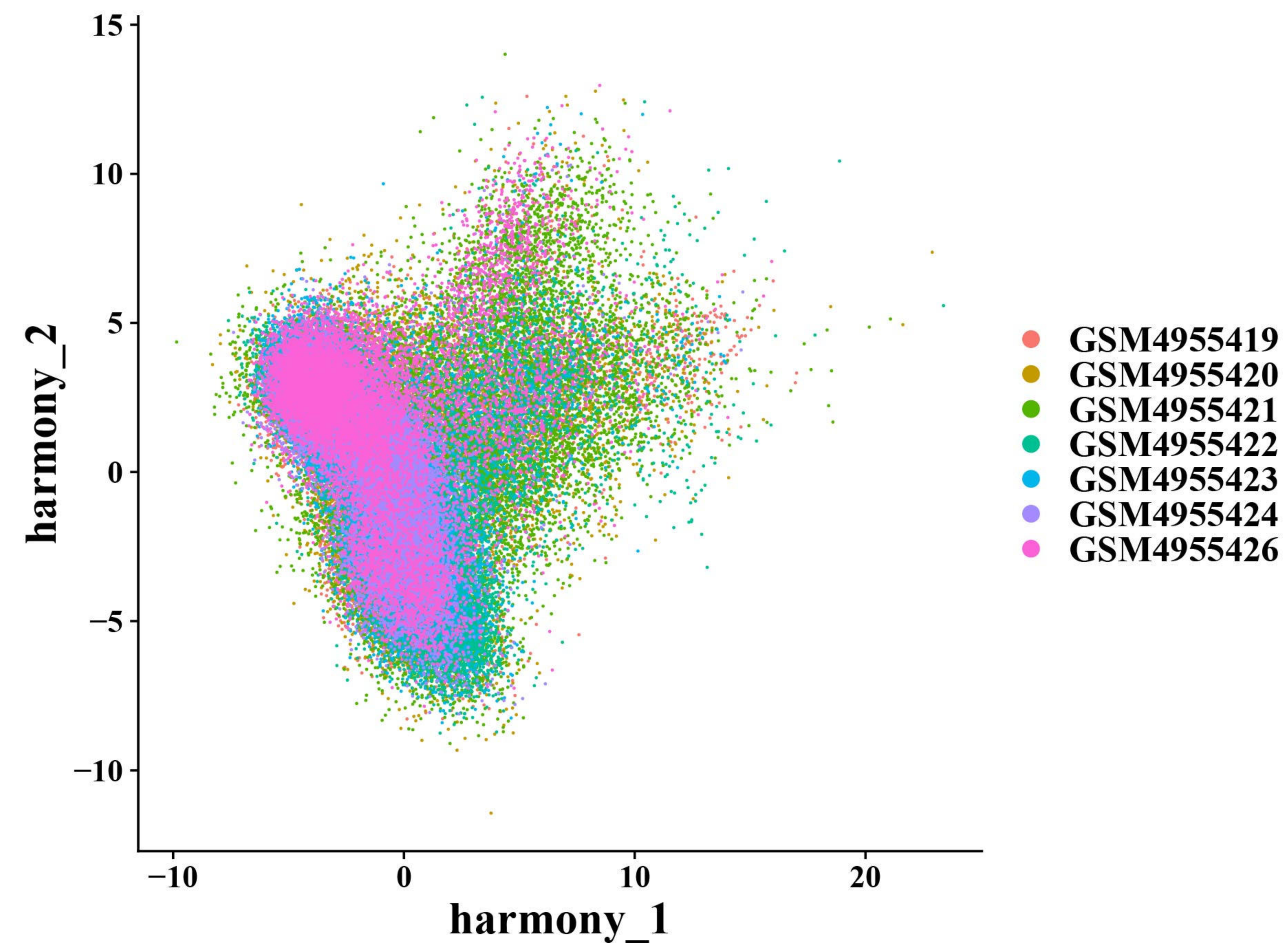

Supplement: Supplementary file 3 — Supporting Information 3 Figure S3: Principal component analysis (PCA) scree plot and selection of the first 20 principal components. [file HUMU-2026-7446280-s003.pdf]

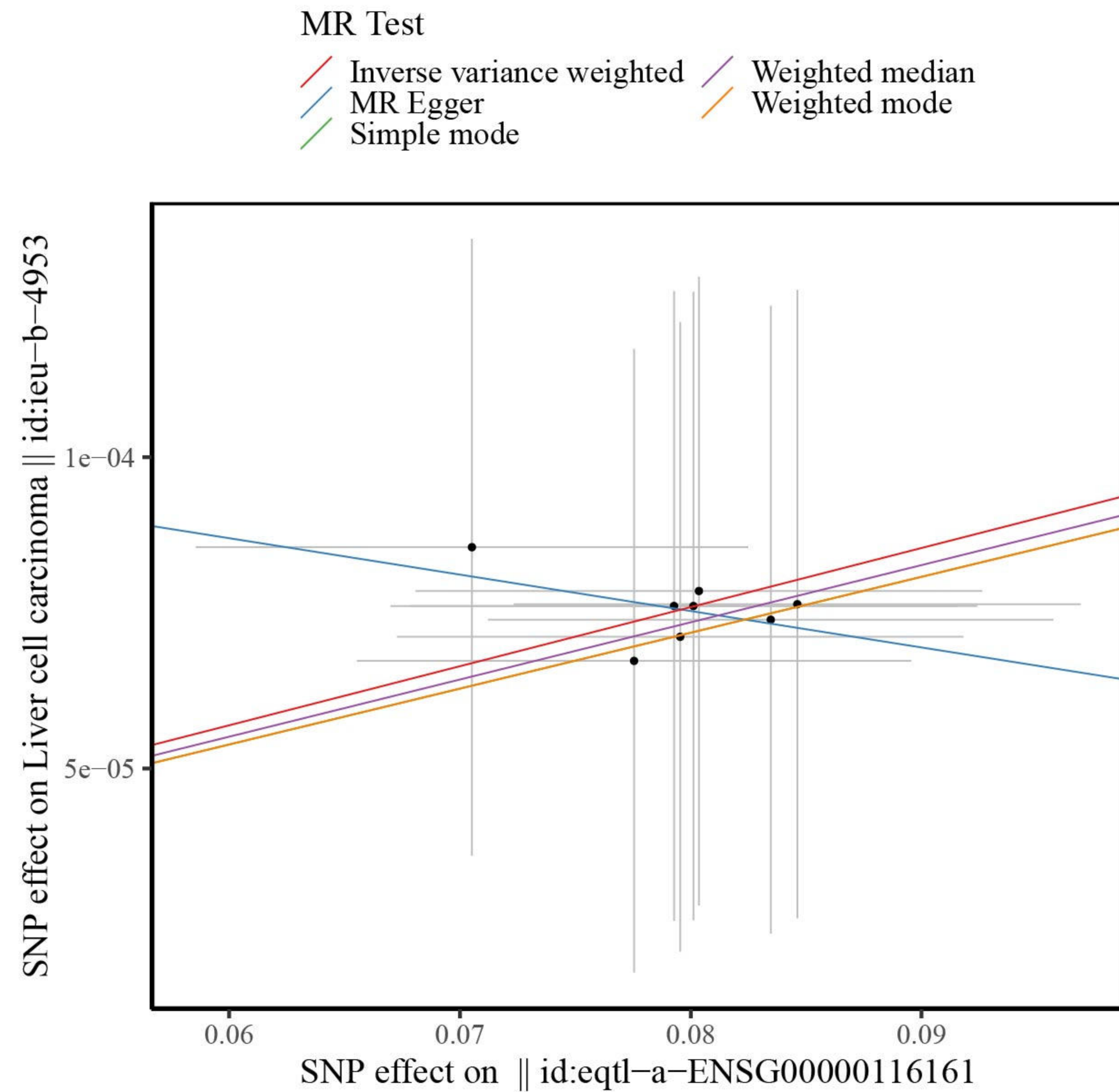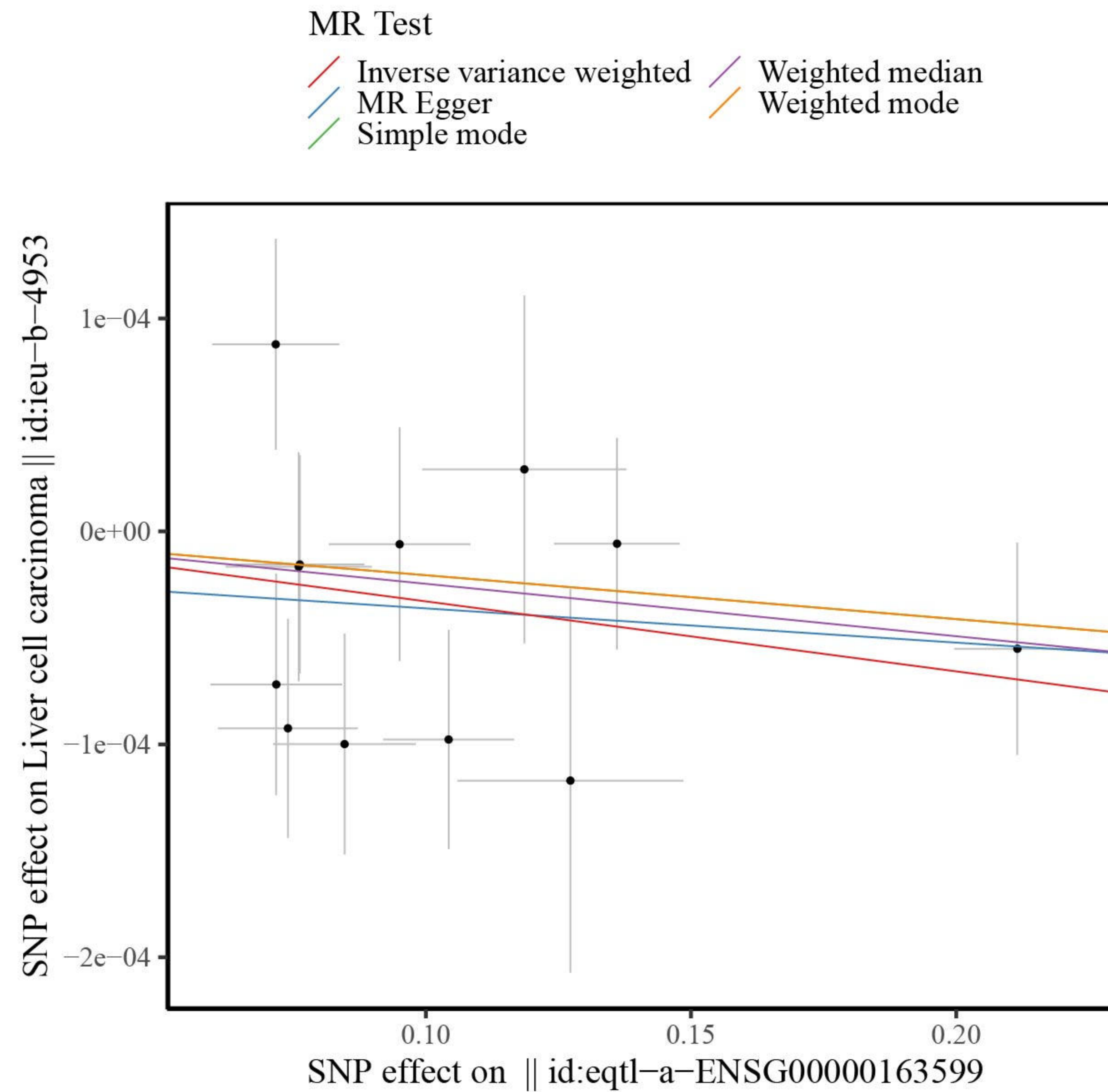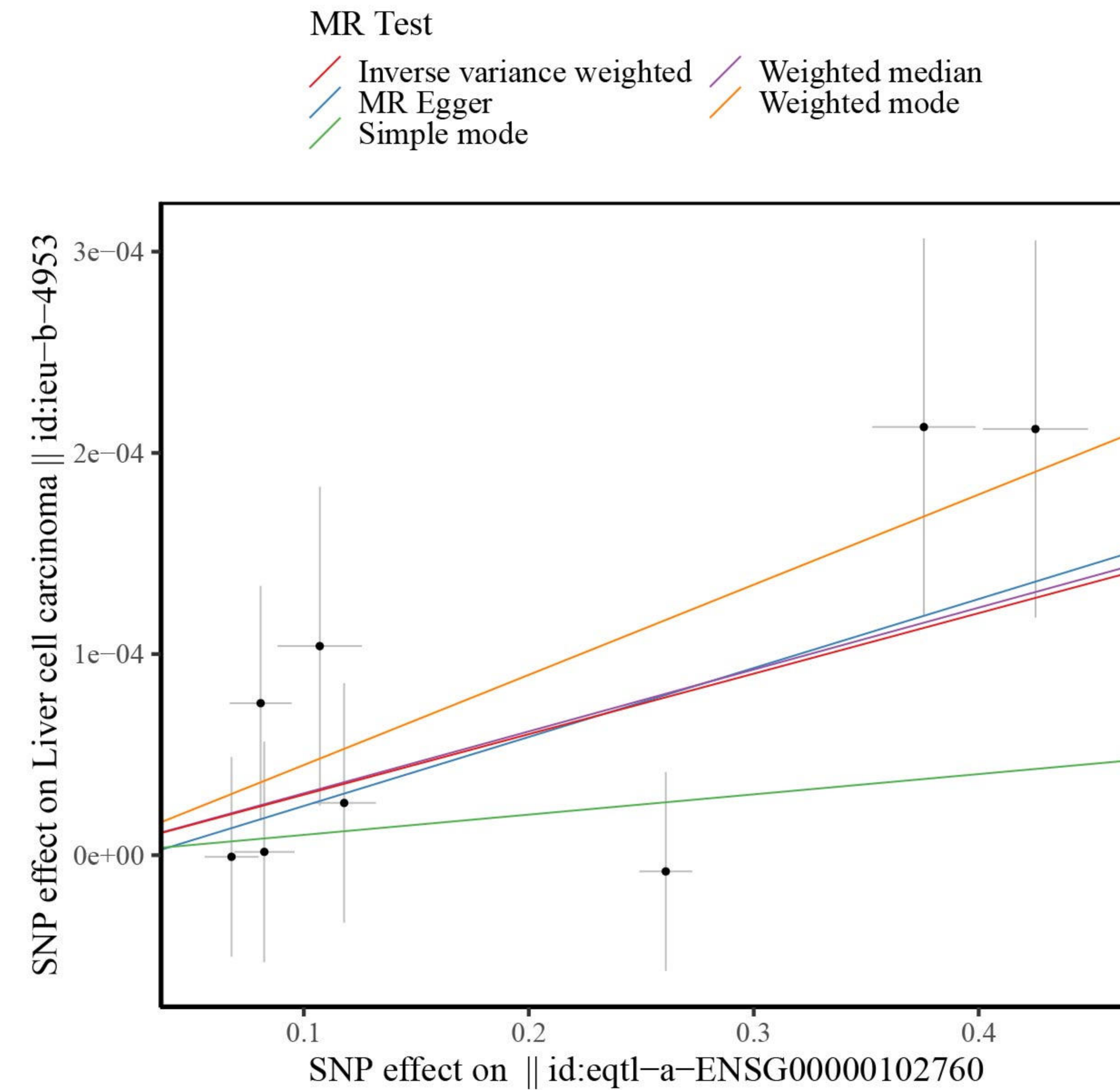

Supplement: Supplementary file 4 — Supporting Information 4 Figure S4: Scatter plots illustrating the causal associations of CACYBP, CTLA4, and RGCC with hepatocellular carcinoma (HCC) in Mendelian randomization analysis. [file HUMU-2026-7446280-s004.pdf]

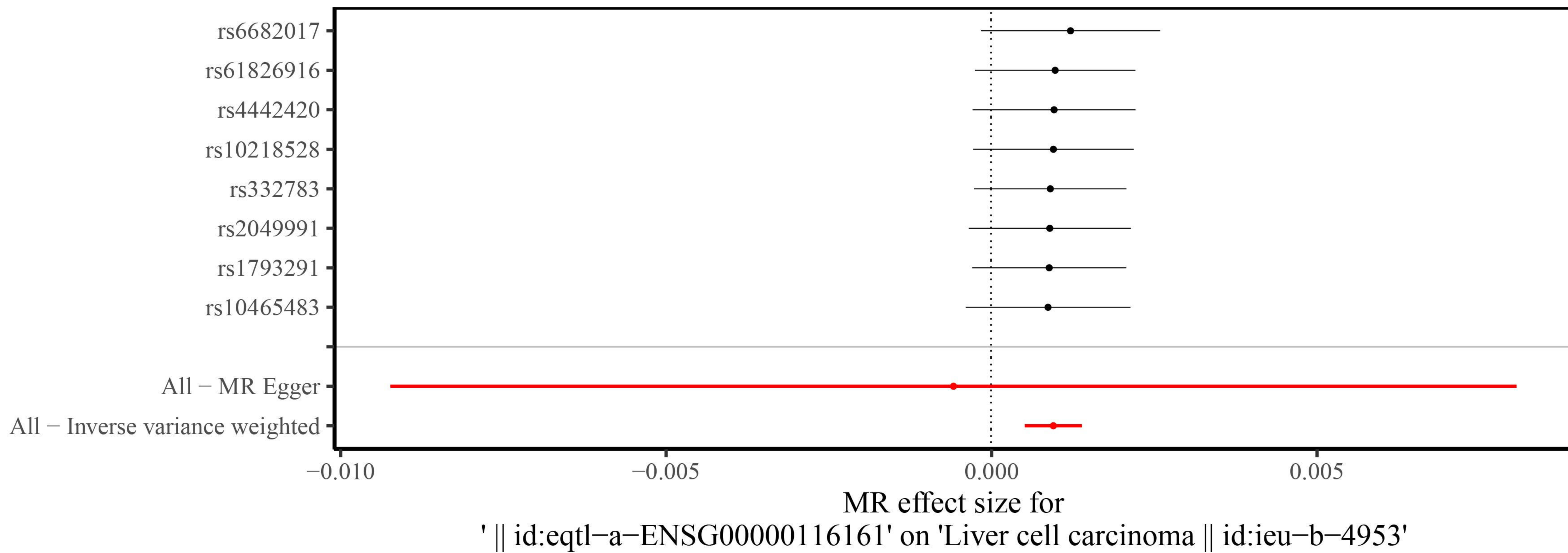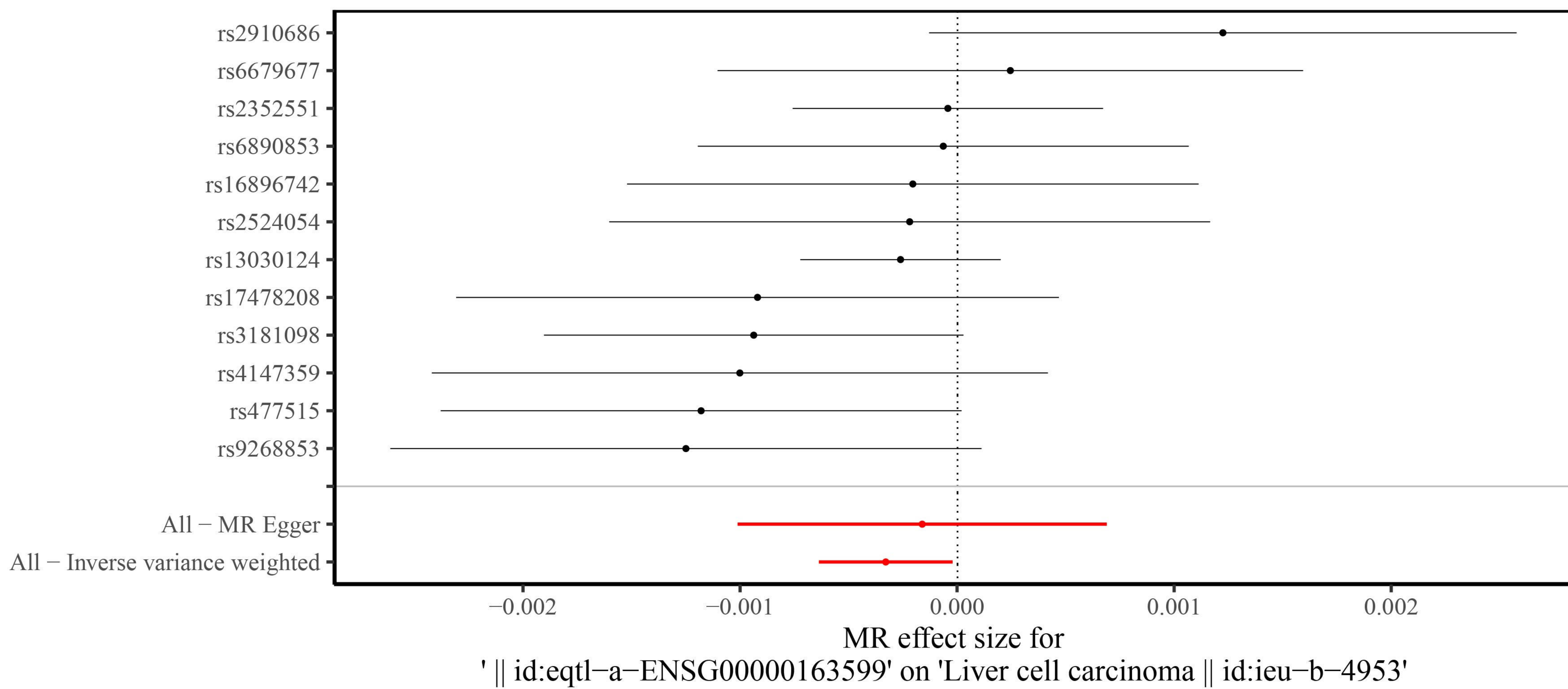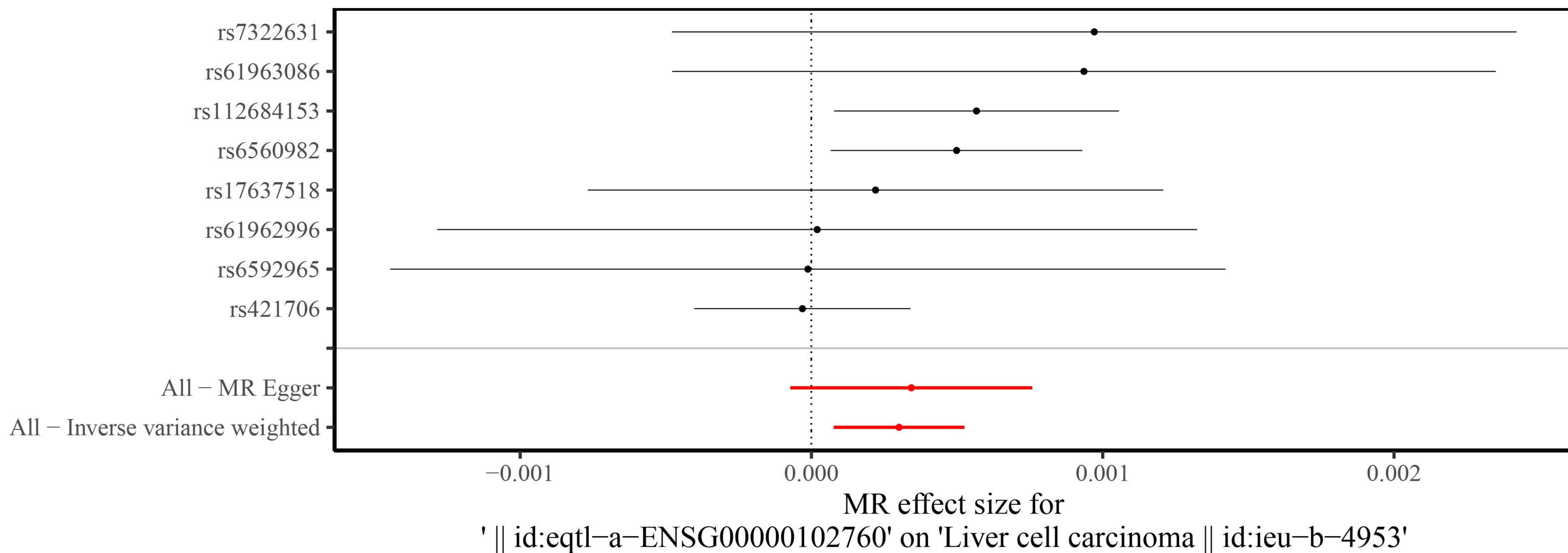

Supplement: Supplementary file 5 — Supporting Information 5 Figure S5: Forest plots showing the effect estimates of instrumental variables for CACYBP, CTLA4, and RGCC on HCC risk using the inverse variance weighted (IVW) method. [file HUMU-2026-7446280-s009.pdf]
