# Supplementary material for: Single‐Cell Sequencing and Mendelian Randomization Reveal T Cell Nuclear Factor Genes in Hepatocellular Carcinoma Progression
Source: Hum Mutat. 2026 Apr 20;2026:7446280. doi: 10.1155/humu/7446280 (PMC13096692; doi:10.1155/humu/7446280)
Supplement: Supplementary file 6 — Supporting Information 6 Figure S6: Funnel plots assessing the symmetry and potential pleiotropy of genetic variants used in Mendelian randomization analysis. [file HUMU-2026-7446280-s006.pdf]

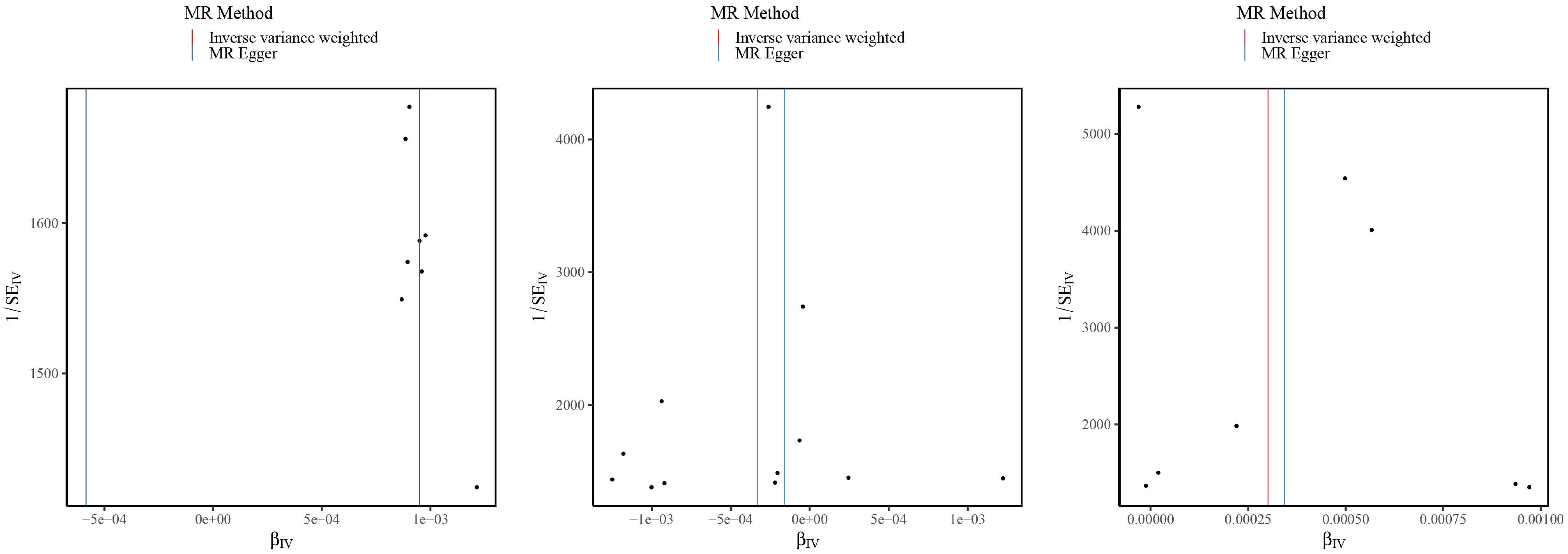
MR Method
Inverse variance weighted
MR Egger
1600
1500
−5e−04
0e+00
5e−04
1e−03
β_{IV}
$1/SE_{IV}$
4000
3000
2000
−1e−03
−5e−04
0e+00
5e−04
1e−03
5000
4000
3000
2000
0.00000
0.00025
0.00050
0.00075
0.00100
