# Supplementary material for: Single‐Cell Sequencing and Mendelian Randomization Reveal T Cell Nuclear Factor Genes in Hepatocellular Carcinoma Progression
Source: Hum Mutat. 2026 Apr 20;2026:7446280. doi: 10.1155/humu/7446280 (PMC13096692; doi:10.1155/humu/7446280)

Integrative Analysis of NFAT-Related Genes in Hepatocellular Carcinoma

Single-Cell Sequencing & Mendelian Randomization Workflow

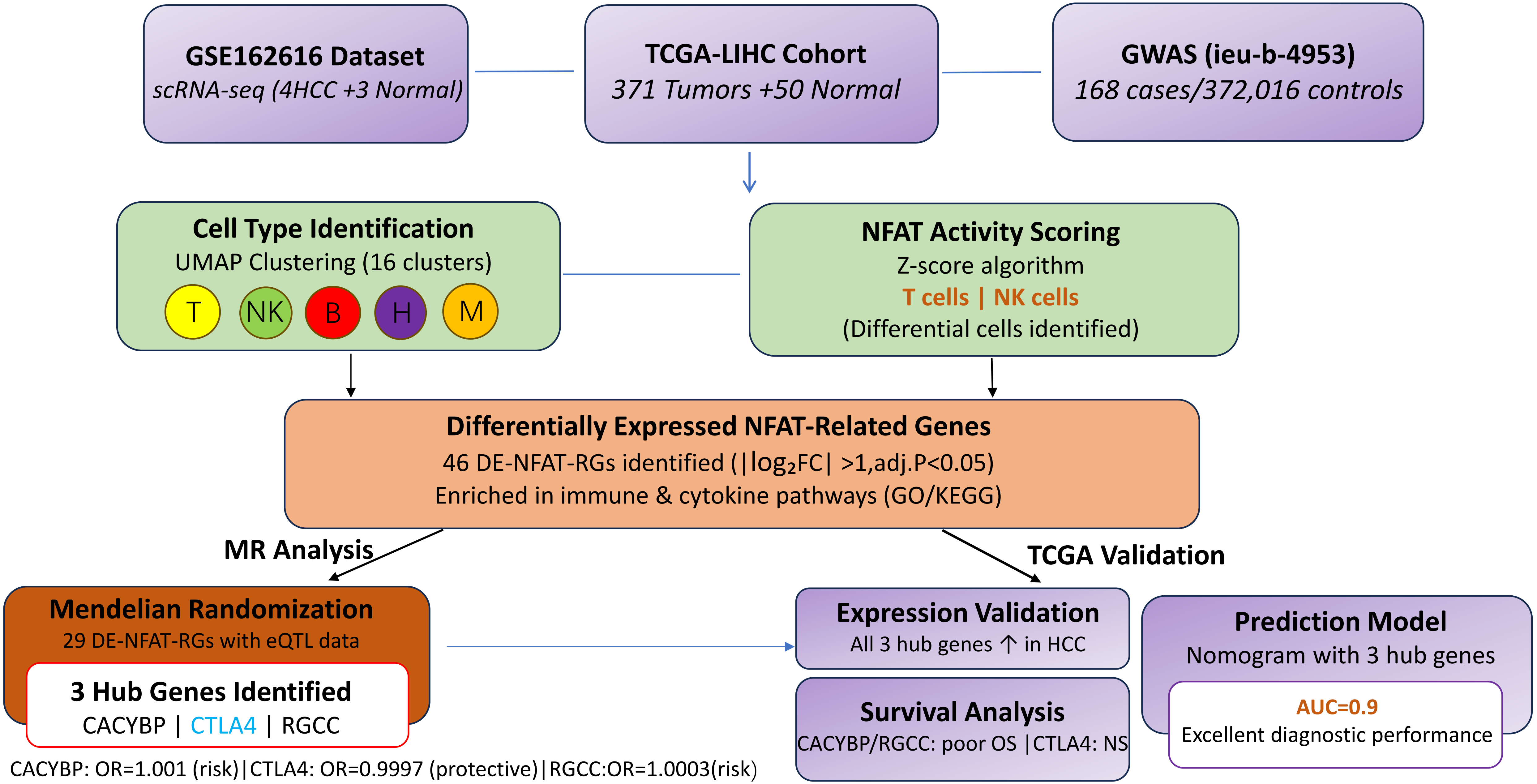

Supplement: Supplementary file 7 — Supporting Information 7 Figure S7: Mechanism schematic diagram. [file HUMU-2026-7446280-s007.pdf]
