# Supplementary material for: Single‐Cell Sequencing and Mendelian Randomization Reveal T Cell Nuclear Factor Genes in Hepatocellular Carcinoma Progression
Source: Hum Mutat. 2026 Apr 20;2026:7446280. doi: 10.1155/humu/7446280 (PMC13096692; doi:10.1155/humu/7446280)
Supplement: Supplementary file 9 — Supporting Information 9 STROBE‐MR Checklist: A completed STROBE‐MR checklist is provided. [file HUMU-2026-7446280-s005.docx]

**STROBE-MR checklist of recommended items to address in reports of Mendelian randomization studies**^1^ ^2^

| **Item No.** | **Section** | **Checklist item** | **Page No.** | **Relevant text from manuscript** |
| --- | --- | --- | --- | --- |
| 1 | **TITLE and ABSTRACT** | Indicate Mendelian randomization (MR) as the study’s design in the title and/or the abstract if that is a main purpose of the study | 1-2 | Single-cell sequencing and Mendelian randomization reveal T cell nuclear factor genes in hepatocellular carcinoma progression  This study uses single-cell RNA sequencing and Mendelian randomization to explore the role of Nuclear Factor of Activated T cells-related genes in hepatocellular carcinoma progression. The GSE162616 dataset was analyzed to identify differential cells and Nuclear Factor of Activated T cells-related genes using quality control, clustering, and z-score algorithms. Mendelian randomization, with expression quantitative trait loci data, identified hub genes in hepatocellular carcinoma The Cancer Genome Atlas-Liver Hepatocellular Carcinoma data was used for validation, survival analysis, and clinical parameter associations, leading to the development of a nomogram for predictive accuracy. |
|  | **INTRODUCTION** |  |  |  |
| 2 | **Background** | Explain the scientific background and rationale for the reported study. What is the exposure? Is a potential causal relationship between exposure and outcome plausible? Justify why MR is a helpful method to address the study question | 3-4 | Mendelian randomization (MR) is predominantly utilized to infer causal relationships between exposure factors and outcomes. Most MR studies employ genetic variants significantly associated with the exposure factor—single nucleotide polymorphisms (SNPs)—as instrumental variables to evaluate the relationship between exposure and outcome. Since genetic variations are randomly inherited from parents at the time of conception, they are less likely to be influenced by potential confounding factors and reverse causality 14,15. MR-based research can further elucidate the causal relationship between exposure factors and outcome variables, offering a more reliable estimate of causal effects than randomized controlled trials. |
| 3 | **Objectives** | State specific objectives clearly, including pre-specified causal hypotheses (if any). State that MR is a method that, under specific assumptions, intends to estimate causal effects | 4 | In light of this, based on the genetic score of NFAT, using NK cells and T cells as the differential cells in the study, and employing Mendelian Randomization (MR) methods, we strive to identify key downstream differential genes. The objective is to furnish references for the diagnosis and management of HCC along with its associated symptoms. |
|  | **METHODS** |  |  |  |
| 4 | **Study design and data sources** | Present key elements of the study design early in the article. Consider including a table listing sources of data for all phases of the study. For each data source contributing to the analysis, describe the following: |  |  |
|  | a) | Setting: Describe the study design and the underlying population, if possible. Describe the setting, locations, and relevant dates, including periods of recruitment, exposure, follow-up, and data collection, when available. | 8-9 | 5 Nuclear factor of activated T-cells related genes (NFAT-RGs) [NFAT1 (NFATp or NFATc2), NFAT2 (NFATc or NFATc1), NFAT3 (NFATc4), NFAT4 (NFATx or NFATc3), and NFAT5 (TonEBP)] were obtained from previous literature 9.  We computed scores for the NFAT-RGs based on the GSE162616 single-cell dataset, which consists of 9 samples. We employed the z-score algorithm from the GSVA package (v 4.1.0) 19 to calculate these scores. Subsequently, the scores were compared between HCC and Normal samples for each cell type using the Wilcoxon test. Cells exhibiting significant score differences between HCC and Normal were identified as differential cells. Finally, the FindMarkers function was used to identify genes that were differentially present in the differential cells, defined as DE-NFAT-RGs (\|log2FC\| > 1 and P.adj < 0.05, and expressed in at least 25% of the cells of a cell population). |
|  | b) | Participants: Give the eligibility criteria, and the sources and methods of selection of participants. Report the sample size, and whether any power or sample size calculations were carried out prior to the main analysis | 10 | We retrieved GWAS data for expression quantitative trait loci (eQTL) of DE-NFAT-RGs and GWAS data of HCC (ieu-b-4953) from the Integrative Epidemiology Unit (IEU) Open genome-wide association study (GWAS) database (https://gwas.mrcieu.ac.uk/). The eQTL summary-level statistics were obtained from the CAGE study, which included 2,765 individuals (mostly of European ancestry) and measured gene expression levels in peripheral blood 21. The ieu-b-4953 included 6,304,034 Single Nucleotide Polymorphism (SNP) from 372,184 samples (HCC: 168, Normal: 372,016). We used DE-NFAT-RGs as the exposure factors and HCC as outcome. |
|  | c) | Describe measurement, quality control and selection of genetic variants | 10 | The extract_instruments function of TwoSampleMR package (v 0.5.6) 22 was used to read exposure factors and screen out SNPs with significant correlation with exposure factors (P < 5*10-8). When clump=TRUE, SNPs with linkage disequilibrium (LD) were removed by setting r2 = 0.001 and kb = 1,000, and we calculated the F-statistic and considered SNPs sufficiently robust when F > 10. The resting SNPs uncorrelated with outcome were acquired in MR analysis. Harmonisation of effect alleles and effect sizes through theharmonise_data function of TwoSampleMR package. The MR methods were MR-Egger 23, Weighted median 24, Inverse variance weighted (IVW) 25, Simple mode 22 and Weighted mode 26. And most important method was IVW due to its better performance to detect causation. |
|  | d) | For each exposure, outcome, and other relevant variables, describe methods of assessment and diagnostic criteria for diseases | 10 | The extract_instruments function of TwoSampleMR package (v 0.5.6) 22 was used to read exposure factors and screen out SNPs with significant correlation with exposure factors (P < 5*10-8). When clump=TRUE, SNPs with linkage disequilibrium (LD) were removed by setting r2 = 0.001 and kb = 1,000, and we calculated the F-statistic and considered SNPs sufficiently robust when F > 10. The resting SNPs uncorrelated with outcome were acquired in MR analysis. Harmonisation of effect alleles and effect sizes through theharmonise_data function of TwoSampleMR package. The MR methods were MR-Egger 23, Weighted median 24, Inverse variance weighted (IVW) 25, Simple mode 22 and Weighted mode 26. And most important method was IVW due to its better performance to detect causation. |
|  | e) | Provide details of ethics committee approval and participant informed consent, if relevant |  |  |
| 5 | **Assumptions** | Explicitly state the three core IV assumptions for the main analysis (relevance, independence and exclusion restriction) as well assumptions for any additional or sensitivity analysis | 10 | Three basic premises underlie MR studies: (1) a robust and significant correlation existed between SNPs and exposure; (2) SNPs were unrelated to confounding factors; (3) SNPs could only influence outcomes through exposure and not through other channels. |
| 6 | **Statistical methods: main analysis** | Describe statistical methods and statistics used |  |  |
|  | a) | Describe how quantitative variables were handled in the analyses (i.e., scale, units, model) | 10 | The extract_instruments function of TwoSampleMR package (v 0.5.6) 22 was used to read exposure factors and screen out SNPs with significant correlation with exposure factors (P < 5*10-8). When clump=TRUE, SNPs with linkage disequilibrium (LD) were removed by setting r2 = 0.001 and kb = 1,000, and we calculated the F-statistic and considered SNPs sufficiently robust when F > 10. The resting SNPs uncorrelated with outcome were acquired in MR analysis. Harmonisation of effect alleles and effect sizes through theharmonise_data function of TwoSampleMR package. The MR methods were MR-Egger 23, Weighted median 24, Inverse variance weighted (IVW) 25, Simple mode 22 and Weighted mode 26. And most important method was IVW due to its better performance to detect causation. |
|  | b) | Describe how genetic variants were handled in the analyses and, if applicable, how their weights were selected | 10 | The extract_instruments function of TwoSampleMR package (v 0.5.6) 22 was used to read exposure factors and screen out SNPs with significant correlation with exposure factors (P < 5*10-8). When clump=TRUE, SNPs with linkage disequilibrium (LD) were removed by setting r2 = 0.001 and kb = 1,000, and we calculated the F-statistic and considered SNPs sufficiently robust when F > 10. The resting SNPs uncorrelated with outcome were acquired in MR analysis. Harmonisation of effect alleles and effect sizes through theharmonise_data function of TwoSampleMR package. The MR methods were MR-Egger 23, Weighted median 24, Inverse variance weighted (IVW) 25, Simple mode 22 and Weighted mode 26. And most important method was IVW due to its better performance to detect causation. |
|  | c) | Describe the MR estimator (e.g. two-stage least squares, Wald ratio) and related statistics. Detail the included covariates and, in case of two-sample MR, whether the same covariate set was used for adjustment in the two samples | 11 | Additionally, Steiger directional test 29 was performed to investigate the presence of reverse causation. Particularly, the genes that passed the reverse causality test were used for subsequent analysis and were selected as hub genes (P < 0.05). |
|  | d) | Explain how missing data were addressed |  |  |
|  | e) | If applicable, indicate how multiple testing was addressed |  |  |
| 7 | **Assessment of assumptions** | Describe any methods or prior knowledge used to assess the assumptions or justify their validity | 10 | To explore DE-NFAT-RGs causally associated with HCC, we conducted a MR analysis.The MR methods were MR-Egger 23, Weighted median 24, Inverse variance weighted (IVW) 25, Simple mode 22 and Weighted mode 26. And most important method was IVW due to its better performance to detect causation. Odds Ratio (OR) > 1 represented risk factor and OR < 1 was protective factor. |
| 8 | **Sensitivity analyses and additional analyses** | Describe any sensitivity analyses or additional analyses performed (e.g. comparison of effect estimates from different approaches, independent replication, bias analytic techniques, validation of instruments, simulations) | 11 | Moreover, sensitivity analysis was executed to evaluate reliability of MR results. Initially, heterogeneity was performed using Cochran's Q test, in which P > 0.05 indicated no heterogeneity 27. Subsequently, horizontal pleiotropy test was carried out to determine the presence of confounding factors via mr_pleiotropy_test function and P > 0.05 indicated the absence of confounding factors in the study 28. Eventually, Leave-one-out (LOO) sensitivity test was conducted by gradually eliminating each SNP to assess the impact of remaining SNPs on the outcome. |
| 9 | **Software and pre-registration** |  |  |  |
|  | a) | Name statistical software and package(s), including version and settings used | 10 | TwoSampleMR package (v 0.5.6) |
|  | b) | State whether the study protocol and details were pre-registered (as well as when and where) |  |  |
|  | **RESULTS** |  |  |  |
| 10 | **Descriptive data** |  |  |  |
|  | a) | Report the numbers of individuals at each stage of included studies and reasons for exclusion. Consider use of a flow diagram |  |  |
|  | b) | Report summary statistics for phenotypic exposure(s), outcome(s), and other relevant variables (e.g. means, SDs, proportions) | 5 | Subsequently, we conducted MR analysis to identify DE-NFAT-RGs with causal association to HCC. A total of 29 DE-NFAT-RGs were screened to obtain SNPs for MR analysis. As a result, in total 3 genes (CACYBP, CTLA4, and RGCC) were causally associated with HCC. |
|  | c) | If the data sources include meta-analyses of previous studies, provide the assessments of heterogeneity across these studies |  |  |
|  | d) | For two-sample MR:  i.  Provide justification of the similarity of the genetic variant-exposure associations between the exposure and outcome samples  ii.  Provide information on the number of individuals who overlap between the exposure and outcome studies | 5 | A total of 29 DE-NFAT-RGs were screened to obtain SNPs for MR analysis. As a result, in total 3 genes (CACYBP, CTLA4, and RGCC) were causally associated with HCC. Thereinto, only CTLA4 [OR = 0.99967, 95% confidence interval (CI): 0.99936-0.99998, P = 0.0363] was associated with decreased risk of HCC, while CACYBP (OR = 1.001, 95% CI: 1.0005-1.0013, P = 2.21e-05), RGCC (OR = 1.0003, 95% CI: 1.000076451-1.000525228, P = 0.0086) were associated with increased risk of HCC (Figure 4a). |
| 11 | **Main results** |  |  |  |
|  | a) | Report the associations between genetic variant and exposure, and between genetic variant and outcome, preferably on an interpretable scale | 5-6 | A total of 29 DE-NFAT-RGs were screened to obtain SNPs for MR analysis. As a result, in total 3 genes (CACYBP, CTLA4, and RGCC) were causally associated with HCC. Thereinto, only CTLA4 [OR = 0.99967, 95% confidence interval (CI): 0.99936-0.99998, P = 0.0363] was associated with decreased risk of HCC, while CACYBP (OR = 1.001, 95% CI: 1.0005-1.0013, P = 2.21e-05), RGCC (OR = 1.0003, 95% CI: 1.000076451-1.000525228, P = 0.0086) were associated with increased risk of HCC (Figure 4a). Specifically, the scatter plots showed the passive correlation of CACYBP, CTLA4, and RGCC with HCC (Fig. S4), the forest plots showed a significant effect of IVW model (Fig. S5), and funnel plots showed that MR analysis conformed to Mendel's second law of random grouping (Fig. S6). To assess the reliability of the MR analysis, a sensitivity analysis was performed. Detailedly, the Cochran's Q test revealed that there was no heterogeneity (P > 0.05) among samples (Table 1). And in horizontal pleiotropy test, there were no confounding factors in the study (P > 0.05) (Table 2). After each SNP was gradually eliminated, the effect of the remaining SNPs on the outcome variables was not significantly changed, indicating that the results of MR analysis were reliable (Figure 4b). Notably, the Steiger directional test revealed that CACYBP, CTLA4, and RGCC with causal association to HCC were used for subsequent analysis (correct_causal_direction=1 and steiger_test_adj<0.05) (Table S1). Accordingly, CACYBP, CTLA4, and RGCC, which were causally associated with HCC, were identified as hub genes in this study. |
|  | b) | Report MR estimates of the relationship between exposure and outcome, and the measures of uncertainty from the MR analysis, on an interpretable scale, such as odds ratio or relative risk per SD difference | 5 | A total of 29 DE-NFAT-RGs were screened to obtain SNPs for MR analysis. As a result, in total 3 genes (CACYBP, CTLA4, and RGCC) were causally associated with HCC. Thereinto, only CTLA4 [OR = 0.99967, 95% confidence interval (CI): 0.99936-0.99998, P = 0.0363] was associated with decreased risk of HCC, while CACYBP (OR = 1.001, 95% CI: 1.0005-1.0013, P = 2.21e-05), RGCC (OR = 1.0003, 95% CI: 1.000076451-1.000525228, P = 0.0086) were associated with increased risk of HCC (Figure 4a). Specifically, the scatter plots showed the passive correlation of CACYBP, CTLA4, and RGCC with HCC (Fig. S4), the forest plots showed a significant effect of IVW model (Fig. S5), and funnel plots showed that MR analysis conformed to Mendel's second law of random grouping (Fig. S6). |
|  | c) | If relevant, consider translating estimates of relative risk into absolute risk for a meaningful time period |  |  |
|  | d) | Consider plots to visualize results (e.g. forest plot, scatterplot of associations between genetic variants and outcome versus between genetic variants and exposure) | 5 | Specifically, the scatter plots showed the passive correlation of CACYBP, CTLA4, and RGCC with HCC (Fig. S4), the forest plots showed a significant effect of IVW model (Fig. S5), and funnel plots showed that MR analysis conformed to Mendel's second law of random grouping (Fig. S6). |
| 12 | **Assessment of assumptions** |  |  |  |
|  | a) | Report the assessment of the validity of the assumptions | 5 | Specifically, the scatter plots showed the passive correlation of CACYBP, CTLA4, and RGCC with HCC (Fig. S4), the forest plots showed a significant effect of IVW model (Fig. S5), and funnel plots showed that MR analysis conformed to Mendel's second law of random grouping (Fig. S6). |
|  | b) | Report any additional statistics (e.g., assessments of heterogeneity across genetic variants, such as *I^2^*, Q statistic or E-value) | 6 | The Cochran's Q test revealed that there was no heterogeneity (P > 0.05) among samples (Table 1). And in horizontal pleiotropy test, there were no confounding factors in the study (P > 0.05) (Table 2). After each SNP was gradually eliminated, the effect of the remaining SNPs on the outcome variables was not significantly changed, indicating that the results of MR analysis were reliable (Figure 4b). Notably, the Steiger directional test revealed that CACYBP, CTLA4, and RGCC with causal association to HCC were used for subsequent analysis (correct_causal_direction=1 and steiger_test_adj<0.05) (Table S1). |
| 13 | **Sensitivity analyses and additional analyses** |  |  |  |
|  | a) | Report any sensitivity analyses to assess the robustness of the main results to violations of the assumptions | 6 | The Cochran's Q test revealed that there was no heterogeneity (P > 0.05) among samples (Table 1). And in horizontal pleiotropy test, there were no confounding factors in the study (P > 0.05) (Table 2). After each SNP was gradually eliminated, the effect of the remaining SNPs on the outcome variables was not significantly changed, indicating that the results of MR analysis were reliable (Figure 4b). Notably, the Steiger directional test revealed that CACYBP, CTLA4, and RGCC with causal association to HCC were used for subsequent analysis (correct_causal_direction=1 and steiger_test_adj<0.05) (Table S1). |
|  | b) | Report results from other sensitivity analyses or additional analyses | 6 | The Cochran's Q test revealed that there was no heterogeneity (P > 0.05) among samples (Table 1). And in horizontal pleiotropy test, there were no confounding factors in the study (P > 0.05) (Table 2). After each SNP was gradually eliminated, the effect of the remaining SNPs on the outcome variables was not significantly changed, indicating that the results of MR analysis were reliable (Figure 4b). Notably, the Steiger directional test revealed that CACYBP, CTLA4, and RGCC with causal association to HCC were used for subsequent analysis (correct_causal_direction=1 and steiger_test_adj<0.05) (Table S1). |
|  | c) | Report any assessment of direction of causal relationship (e.g., bidirectional MR) | 6 | Notably, the Steiger directional test revealed that CACYBP, CTLA4, and RGCC with causal association to HCC were used for subsequent analysis (correct_causal_direction=1 and steiger_test_adj<0.05) (Table S1). |
|  | d) | When relevant, report and compare with estimates from non-MR analyses | 5 | Thereinto, only CTLA4 [OR = 0.99967, 95% confidence interval (CI): 0.99936-0.99998, P = 0.0363] was associated with decreased risk of HCC, while CACYBP (OR = 1.001, 95% CI: 1.0005-1.0013, P = 2.21e-05), RGCC (OR = 1.0003, 95% CI: 1.000076451-1.000525228, P = 0.0086) were associated with increased risk of HCC (Figure 4a). Specifically, the scatter plots showed the passive correlation of CACYBP, CTLA4, and RGCC with HCC (Fig. S4), the forest plots showed a significant effect of IVW model (Fig. S5), and funnel plots showed that MR analysis conformed to Mendel's second law of random grouping (Fig. S6). |
|  | e) | Consider additional plots to visualize results (e.g., leave-one-out analyses) | 6 | After each SNP was gradually eliminated, the effect of the remaining SNPs on the outcome variables was not significantly changed, indicating that the results of MR analysis were reliable (Figure 4b). |
|  | **DISCUSSION** |  |  |  |
| 14 | **Key results** | Summarize key results with reference to study objectives | 18-19 | This study integrates single-cell RNA sequencing and Mendelian randomization to identify CACYBP, CTLA4, and RGCC as causal hub genes in hepatocellular carcinoma (HCC), with elevated expression in tumors. T cells and Natural Killer cells were highlighted as central to the tumor immune microenvironment, and a diagnostic nomogram was developed based on these genes. These findings offer insights into immune-related mechanisms and potential targets for HCC immunotherapy. |
| 15 | **Limitations** | Discuss limitations of the study, taking into account the validity of the IV assumptions, other sources of potential bias, and imprecision. Discuss both direction and magnitude of any potential bias and any efforts to address them | 18 | However, this study still faces the issue of a small sample size, and the connection and mechanism of action of the three hub genes, especially RGCC, in the development and progression of HCC, need to be further confirmed through additional exploration. It is believed that as our exploration of the tumor immune microenvironment continues, the clinical treatment system for HCC will be continuously refined. |
| 16 | **Interpretation** |  |  |  |
|  | a) | Meaning: Give a cautious overall interpretation of results in the context of their limitations and in comparison with other studies | 18 | We have established a nomogram prediction model and scoring system using the aforementioned three hub genes as risk factors, with the occurrence of Hepatocellular Carcinoma (HCC) as the outcome variable. We found that CACYBP has the largest proportion in the occurrence probability of HCC, This indicates that the expression level of CACYBP plays an absolutely dominant role in this predictive model, while RGCC and CTLA4 may have certain variability. This predictive model uses the expression levels of the three hub genes as independent variables, is relatively simple to operate, and has a significantly lower cost compared to other multi-gene predictive models. It is fully applicable for clinical prediction of HCC occurrence and evaluation of treatment effects, thus holding significant potential for clinical translation. |
|  | b) | Mechanism: Discuss underlying biological mechanisms that could drive a potential causal relationship between the investigated exposure and the outcome, and whether the gene-environment equivalence assumption is reasonable. Use causal language carefully, clarifying that IV estimates may provide causal effects only under certain assumptions | 13 | Based on the genetic score of NFAT, we used NK cells and T cells as differentiated cells, and adopted Mendelian randomization (MR) method to find that three hub genes (CACYBP, CTLA4, RGCC) significantly affected HCC. |
|  | c) | Clinical relevance: Discuss whether the results have clinical or public policy relevance, and to what extent they inform effect sizes of possible interventions | 18 | We have established a nomogram prediction model and scoring system using the aforementioned three hub genes as risk factors, with the occurrence of Hepatocellular Carcinoma (HCC) as the outcome variable. We found that CACYBP has the largest proportion in the occurrence probability of HCC, This indicates that the expression level of CACYBP plays an absolutely dominant role in this predictive model, while RGCC and CTLA4 may have certain variability. This predictive model uses the expression levels of the three hub genes as independent variables, is relatively simple to operate, and has a significantly lower cost compared to other multi-gene predictive models. It is fully applicable for clinical prediction of HCC occurrence and evaluation of treatment effects, thus holding significant potential for clinical translation. |
| 17 | **Generalizability** | Discuss the generalizability of the study results (a) to other populations, (b) across other exposure periods/timings, and (c) across other levels of exposure |  |  |
|  | **OTHER INFORMATION** |  |  |  |
| 18 | **Funding** | Describe sources of funding and the role of funders in the present study and, if applicable, sources of funding for the databases and original study or studies on which the present study is based | 19 | The study was supported by the Project of Science & Technology Department of Sichuan Province key R & D plan (grant number:23ZDYF2443), the Research Project Plan of Sichuan Medical Association(grant number: S210018). |
| 19 | **Data and data sharing** | Provide the data used to perform all analyses or report where and how the data can be accessed, and reference these sources in the article. Provide the statistical code needed to reproduce the results in the article, or report whether the code is publicly accessible and if so, where | 19 | The data that support the findings of this study are openly available in [UCSC] at [http://xena.ucsc.edu/], [GEO] at [https://www.ncbi.nlm.nih.gov/gds] reference number [GSE162616] and [GWAS] at [https://gwas.mrcieu.ac.uk/] reference number [ieu-b-4953]. |
| 20 | **Conflicts of Interest** | All authors should declare all potential conflicts of interest | 19 | The authors declare no competing interests. |

This checklist is copyrighted by the Equator Network under the Creative Commons Attribution 3.0 Unported (CC BY 3.0) license.

1. Skrivankova VW, Richmond RC, Woolf BAR, Yarmolinsky J, Davies NM, Swanson SA, et al. Strengthening the Reporting of Observational Studies in Epidemiology using Mendelian Randomization (STROBE-MR) Statement. JAMA. 2021;under review.

2. Skrivankova VW, Richmond RC, Woolf BAR, Davies NM, Swanson SA, VanderWeele TJ, et al. Strengthening the Reporting of Observational Studies in Epidemiology using Mendelian Randomisation (STROBE-MR): Explanation and Elaboration. BMJ. 2021;375:n2233.
